# Targeting cis-regulatory elements of FOXO family is a novel therapeutic strategy for induction of leukemia cell differentiation

**DOI:** 10.1038/s41419-023-06168-2

**Published:** 2023-09-29

**Authors:** Kenta Kurayoshi, Yusuke Takase, Masaya Ueno, Kumiko Ohta, Kyoko Fuse, Shuji Ikeda, Takayoshi Watanabe, Yuki Nishida, Shin-ichi Horike, Kazuyoshi Hosomichi, Yuichi Ishikawa, Yuko Tadokoro, Masahiko Kobayashi, Atsuko Kasahara, Yongwei Jing, Mahmoud I. Shoulkamy, Makiko Meguro-Horike, Kensuke Kojima, Hitoshi Kiyoi, Hiroshi Sugiyama, Hiroki Nagase, Atsushi Tajima, Atsushi Hirao

**Affiliations:** 1 Division of Molecular Genetics, Cancer Research Institute, Kanazawa University, Kakuma-machi, Kanazawa 920-1192 Japan; 2 Division of Molecular Genetics, WPI Nano Life Science Institute (WPI-Nano LSI), Kanazawa University, Kakuma-machi, Kanazawa 920-1192 Japan; 3 Department of Pharmacy, University of the Ryukyus Hospital, 207 Uehara, Nishihara, Nakagami District, Okinawa 903-0215 Japan; 4 Department of Hematopoietic Cell Transplantation, Niigata University Medical and Dental Hospital, 1-757 Asahimachi-dori Chuoh-ku, Niigata, 951-8510 Japan; 5 Department of Chemistry, Graduate School of Science, Kyoto University, Kitashirakawa-Oiwakecho, Sakyo-ku, Kyoto 606-8502 Japan; 6 Department of Molecular Carcinogenesis, Chiba Cancer Center Research Institute, Chuo-ku, Chiba 260-8717 Japan; 7 Section of Molecular Hematology and Therapy, Department of Leukemia, The University of Texas MD Anderson Cancer Center, Houston, TX 77030 USA; 8 Division of Integrated Omics Research, Research Center for Experimental Modeling of Human Disease Kanazawa University, Kanazawa University, 13-1 Takara-machi, Kanazawa, 920-0934 Japan; 9 Department of Bioinformatics and Genomics, Graduate School of Advanced Preventive Medical Sciences, Kanazawa University, 13-1 Takara-machi, Kanazawa, Ishikawa 920-8640 Japan; 10 Department of Hematology and Oncology, Nagoya University Graduate School of Medicine, 65 Tsurumai-cho, Showa-ku, Nagoya, Aichi 466-8550 Japan; 11 Division of Molecular Genetics, Institute for Frontier Science Initiative, Kanazawa University, Kakuma-machi, Kanazawa, Ishikawa 920-1192 Japan; 12 Zoology Department, Faculty of Science, Minia University, El-Minia, 61519 Egypt; 13 Department of Hematology, Kochi Medical School Hospital, Kochi University, Okocho Kohasu, Nankoku, Kochi 783-8505 Japan; 14 Institute for Integrated Cell-Material Sciences (WPI-iCeMS), Kyoto University, Yoshida-Ushinomaecho, Sakyo-ku, Kyoto 606-8502 Japan; 15 Intractable Disease Research Center, Juntendo University Graduate School of Medicine, 2-1-1 Hongo, Bunkyo-ku, Tokyo 113-8421 Japan; 16 Present Address: Laboratory of Computational Genomics, Tokyo University of Pharmacy and Life Science, 1432-1 Horinouchi, Hachioji, Tokyo 192-0392 Japan

**Keywords:** Mechanisms of disease, Drug development

## Abstract

Differentiation therapy has been proposed as a promising therapeutic strategy for acute myeloid leukemia (AML); thus, the development of more versatile methodologies that are applicable to a wide range of AML subtypes is desired. Although the FOXOs transcription factor represents a promising drug target for differentiation therapy, the efficacy of FOXO inhibitors is limited in vivo. Here, we show that pharmacological inhibition of a common cis-regulatory element of forkhead box O (FOXO) family members successfully induced cell differentiation in various AML cell lines. Through gene expression profiling and differentiation marker-based CRISPR/Cas9 screening, we identified *TRIB1*, a complement of the COP1 ubiquitin ligase complex, as a functional FOXO downstream gene maintaining an undifferentiated status. TRIB1 is direct target of FOXO3 and the FOXO-binding cis-regulatory element in the *TRIB1* promoter, referred to as the FOXO-responsive element in the *TRIB1* promoter (FRE-T), played a critical role in differentiation blockade. Thus, we designed a DNA-binding pharmacological inhibitor of the FOXO-FRE-T interface using pyrrole-imidazole polyamides (PIPs) that specifically bind to FRE-T (FRE-PIPs). The FRE-PIPs conjugated to chlorambucil (FRE-chb) inhibited transcription of *TRIB1*, causing differentiation in various AML cell lines. FRE-chb suppressed the formation of colonies derived from AML cell lines but not from normal counterparts. Administration of FRE-chb inhibited tumor progression in vivo without remarkable adverse effects. In conclusion, targeting cis-regulatory elements of the FOXO family is a promising therapeutic strategy that induces AML cell differentiation.

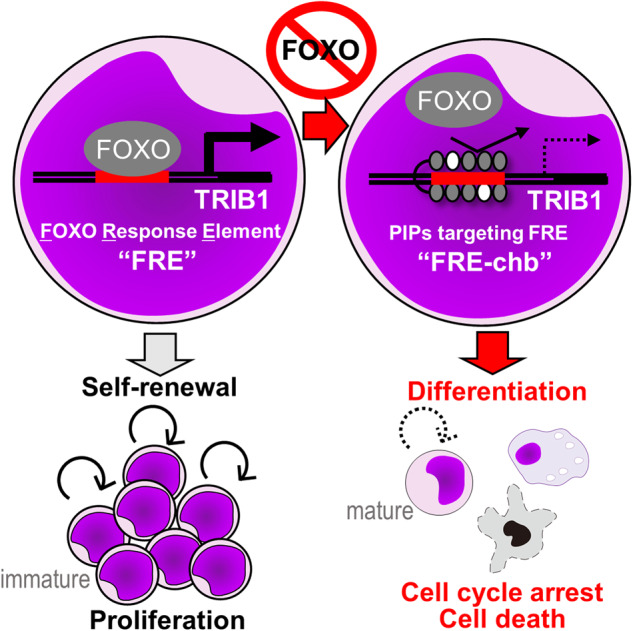

## Introduction

Differentiation blockade in progenitor cells is a critical pathological feature of acute myeloid leukemia (AML). To date, various driver genes that contribute to differentiation blockade have been identified. Acute promyelocytic leukemia is a subtype of AML characterized by the fusion gene, *PML*–*RARA*, which recruits histone deacetylase complexes to a group of genes involved in differentiation. All-trans retinoic acid directly binds to *PML*–*RARA* and promotes its degradation, leading to the induction of differentiation and excellent therapeutic outcomes [1]. Other oncogenes such as metabolic enzymes (isocitrate dehydrogenase 1/2) or epigenetic regulators (lysine-specific histone demethylase 1) contribute to differentiation blockade, and inhibitors of these oncogenes also exhibit successful therapeutic efficacy [2, 3]. Therefore, differentiation therapy represents a promising strategy for AML, and differentiation agents are being developed that target driver genes. However, successfully treated cases using this therapy remain limited. Thus, to further expand the range of indications for differentiation therapy, the development of methodologies that are applicable to a wider range of AML subtypes is desired.

Forkhead box O subclass (FOXO) is a group of transcription factors consisting of FOXO1, FOXO3, FOXO4, and FOXO6. FOXOs are involved in various biological processes such as cell proliferation, apoptosis, and resistance to stress. FOXOs are regulated by phosphorylation through protein kinase B (AKT/PKB), which leads to the nuclear exclusion of FOXOs, resulting in a loss of transcriptional activity. Consistent with the oncogenic role of AKT, FOXO target genes include tumor-suppressive genes (BCL2L11(Bim), BBC3(PUMA), CDKN1B(p27^Kip^)), and overexpression of FOXOs induces apoptosis and cell cycle arrest, while suppression of FOXOs promotes tumorigenesis in prostate, gastric, and thymic tumors [4–9]. Therefore, FOXOs are generally considered to be tumor suppressors. In contrast, we have shown that FOXO3 plays a pivotal role in leukemia stem cells and progression of leukemia [10]. Additionally, FOXOs contribute to chemotherapy resistance in various cancer types including leukemia, breast cancer, and ovarian cancer [11–13]. These observations mean that FOXOs contribute to malignancy in cancer depending on the cellular circumstances and environment. FOXO3 is essential for differentiation blockade in human AML cell lines harboring different types of mutations [14]. Importantly, overexpression of FOXO1 is sufficient to induce differentiation blockade and a preleukemic state in human CD34+ hematopoietic stem and progenitor cells (HSPCs) [15]. These observations suggest that FOXOs are crucial transcription factors that sustain the oncogenic network and undifferentiated status in human AML cell lines. Therefore, targeting FOXOs could represent a promising approach for differentiation therapy. The most commonly used FOXO1 inhibitor, AS1842856, selectively binds to the phosphorylated form (active form) of FOXO1 and predominantly represses its transcriptional activity [16]. Despite its potential in in vivo experiments, it needs to be administered frequently and in high doses for FOXO1 inhibition in vivo [17]. Additionally, although FOXO3 is also a promising therapeutic target, selective FOXO3 inhibitors available for in vivo treatment have not yet been developed. Thus, intense research efforts are underway to develop FOXO1 and FOXO3 inhibitors [18].

The activity of transcription factors is tightly regulated through the fine-tuning of their binding to cofactors and cis-regulatory elements. Generally, transcription factors are considered “undruggable targets” because of their difficulty inhibiting their interactions with small molecule compounds. As a promising strategy for inhibiting transcription factor activity, hairpin pyrrole-imidazole polyamides (PIPs), which are synthetic DNA-binding molecules, have gained increasing attention. Because the pairing of imidazole (Im) against pyrrole (Py) (Im/Py) preferentially binds to a G/C base pair, and a Py/Py pair recognizes an A/T or T/A base pair, PIPs that bind to arbitrary sequences could be created by changing the order of Py/Im and Py/Py [19]. PIPs targeting the cis-regulatory elements could disrupt the transcription factor–DNA interface and suppress the expression of its target gene [20]. PIPs conjugated with an alkylating agent such as chlorambucil (Chb) form covalent bonds against their binding sequence to efficiently reduce their target gene expression [21–23]. Importantly, PIPs have exhibited antitumor effects in various cancer models in vivo [21, 24, 25]. Therefore, they are promising tools for inhibition of FOXOs and in AML therapy. To develop an effective FOXO inhibitor using PIPs, the cis-regulatory element of a functional FOXO target needs to be identified because of the diversity of cis-regulatory elements of FOXOs depending on their target genes. Although intensive research on FOXOs has revealed these target genes, functional FOXO downstream genes maintaining an undifferentiated status remain unidentified. This has also caused uncertainty regarding the mechanism of differentiation blockade by FOXOs.

Here, we identified *tribbles pseudokinase 1* (*TRIB1*), a component of the COP1 ubiquitin ligase complex, as a functional molecule in AS1842856-induced differentiation in AML. The FOXO binding cis-regulatory element in the promoter region of the *TRIB1* gene, referred to as the FOXO-responsive element in the *TRIB1* promoter (FRE-T), plays a critical role in the blockade of AML differentiation. Pharmacological inhibition of a common cis-regulatory element of FOXOs, including FRE-T, using PIPs conjugated to chlorambucil (FRE-chb), successfully induced differentiation in AML. Administration of FRE-chb inhibited tumor progression in vivo without remarkable adverse effects. These data revealed that targeting cis-regulatory elements of the FOXO family is a potent therapeutic strategy for inducing differentiation in AML.

## Materials and methods

### CRISPR library screening

In this study, 30 genes were evaluated through CRISPR library screening to identify genes crucial for AML differentiation blockade. The sequences coding for 1004 non-targeting control sgRNAs and sgRNAs targeting 30 genes were obtained from validated sgRNA libraries published previously [26]. The preparation of the sgRNA pool was performed as described previously [27]. The PCR product was cloned into lentiCRISPRv2 were purchased from Gibson Assembly (NEB). The Gibson Assembly reaction products were transformed into 5-alpha electrocompetent *E. coli* (NEB). Virus pools were produced by co-transfection of the sgRNA-expressing vectors with VSV-G and psPAX2 plasmids at a 1:1:0.5 ratio into HEK-293T cells using X-tremeGENE HP DNA Transfection Reagent (Roche). Genomic DNA samples were extracted and sgRNA inserts were amplified by PCR, and the resultant PCR products were sequenced by MiSeq (Illumina). The results of next-generation sequencing are shown in Table S1. From the sets of FASTQ files, the adapter sequence was removed by FASTX-Toolkit software (https://hannonlab.cshl.edu/fastx_toolkit/), and sequence data were aligned against the reference by BWA software [28]. Each read count of the individual sgRNA sequence was calculated by SAMtools [29]. A bash shell script for this analysis is available from the authors upon reasonable request. *p* values were corrected using MAGeCK [30].

### Xenotransplantation assay

NOD.Cg-Prkdc scid Il2rg tm1Wjl/SzJ (NSG) mice were purchased from NINOX Labo Supply Inc. (Ishikawa, Japan). SKM-1 cells (5 × 10^6^ cells) were suspended in 50 µL of RPMI-1640 prior to being mixed with an additional 50 µL of Matrigel (Corning) and subcutaneously transplanted into 6-week-old female NSG mice. At post-transplantation day 3, the mice were randomly divided into three groups based on body weight and treated with vehicle, Chb-S, or FRE-chb (1.28 mg/kg per intravenous injection every 2 days). Tumor volume was calculated using the formula (long diameter) × (short diameter)^2^/2. During the experiments, the researchers were aware of the group assignments.

### Plasmid construction

1319 pcDNA3 flag FKHRL1 AAA was a gift from William Sellers (Plasmid #10709) [31]. pLKO.1 puro was a gift from Bob Weinberg (Plasmid #8453) [32]. scramble shRNA was a gift from David Sabatini (Plasmid #1864) [33]. pLJM1-Empty was a gift from Joshua Mendell (Plasmid #91980) [34]. lentiCRISPR v2 was a gift from Feng Zhang (Plasmid #52961) [35]. An expression vector for *TRIB1* (pLJM1 TRIB1) was generated by subcloning *TRIB1* from pCR3HA into pLJM1 [36]. The *TRIB1* promoter was amplified from the HL-60 genome and cloned into pGL3 basic (pTRIB1wt-Luc). pTRIB1mt-Luc, in which the FOXO consensus sequence was depleted, was developed by site-directed mutagenesis. Expression vectors for sgRNA and shRNA were constructed by subcloning annealed oligonucleotides into lentiGuide-Puro and pLKO.1 puro, respectively. Target sequences of sgRNA and shRNA are listed in Table S2.

### Statistical analyses

Data are shown as mean ± SD values of the indicated number of independent experiments. The sample size was predetermined based on pertinent literature. Statistical differences between groups were assessed by two-sided Welch’s t-test. A *p* value of <0.05 was considered to be statistically significant. The statistical correlation of FOXO3 and TRIB1 gene expressions in AML patients’ samples were assessed by the Spearman’s rank correlation coefficient.

## Results

### AS1842856 overcomes differentiation blockade in AML cell lines

**Fig. 1:**
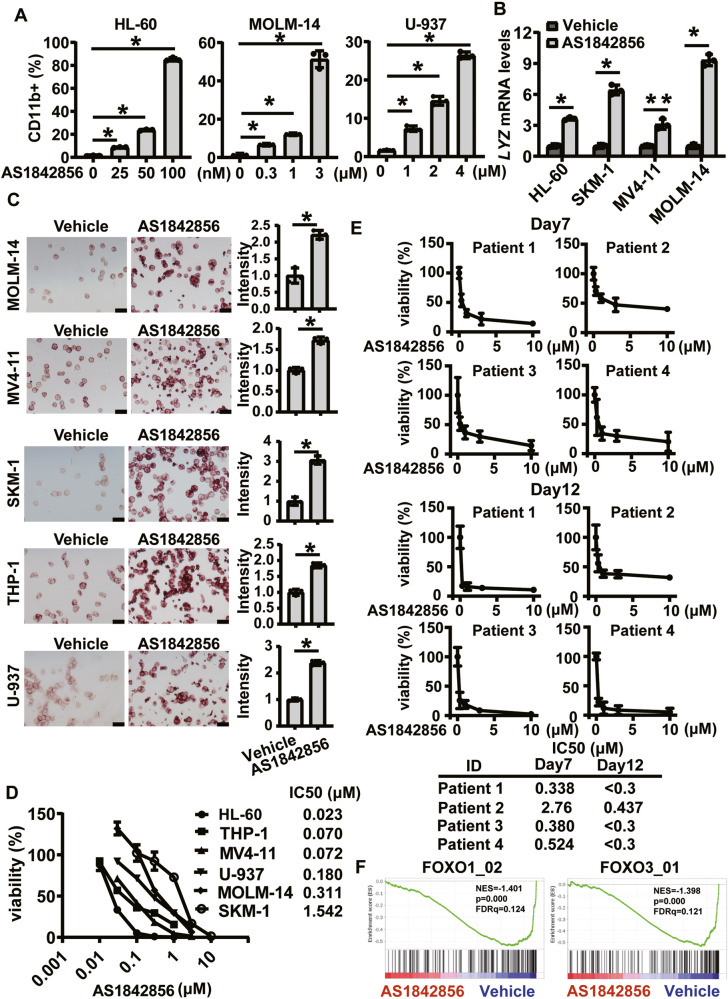
AS1842856 induces differentiation in AML cell lines. **A** FACS analysis of CD11b in HL-60, MOLM-14, and U-937 cells treated with AS1842856 for 4 days. Data are mean ± SD (*n* = 3). *P < 0.01. Two-side Welch’s t-test. **B** RT-qPCR for quantification of *LYZ* expression in individual cell lines treated with AS1842856. HL-60 cells were treated with AS1842856 (100 nM) for 3 days. SKM-1 cells were treated with AS1842856 (1 μM) for 3 days. MV4-11 cells were treated with AS1842856 (4 μM) for 3 days. MOLM-14 cells were treated with AS1842856 (4 μM) for 4 days. Data are mean ± SD (*n* = 3). *P < 0.01, **P < 0.05. Two-side Welch’s t-test. **C** NSE activity of individual cell lines treated with AS1842856. MOLM-14 cells were treated with AS1842856 (4 μM) for 3 days. MV4-11 cells were treated with AS1842856 (4 μM) for 2 days. SKM-1 cells were treated with AS1842856 (1 μM) for 6 days. THP-1 cells were treated with AS1842856 (1 μM) for 5 days. U-937 cells were treated with AS1842856 (2 μM) for 4 days. Data are mean ± SD (*n* = 3). *P < 0.01. Two-side Welch’s t-test. **D** IC50s of AS1842856 evaluated in individual cell lines after treatment with AS1842856 for 6 days. Data are mean ± SD (*n* = 3). **E** Cell viability of primary AML samples after AS1842856 treatment for 7 or 12 Days. Data are mean ± SD (*n* = 3). **F** GSEA enrichment curves of gene sets associated with FOXO1 and FOXO3. NES normalized enrichment score; FDR false discovery rate.

To investigate the potential of FOXOs as druggable targets for differentiation therapy, we evaluated the differentiation phenotypes of six AML cell lines after treatment with AS1842856. We found that AS1842856 induced myeloid markers including CD11b, non-specific esterase (NSE) activity, or lysozyme (*LYZ*) mRNA expression, in all six cell lines (Fig. 1A–C). Additionally, the compound efficiently reduced cell viability at certain concentrations (IC50: 0.023–1.542 μM) (Fig. 1D). AS1842856 was effective in suppressing the viability of primary AML samples, especially after 12 days of treatment (Fig. 1E). These data indicate that this compound induced differentiation in various AML cell lines and inhibited proliferation. Importantly, AS1842856 suppressed genes related to the FOXO family (Fig. 1F). These findings suggest that, although there could be off-target effects, this compound induces differentiation of AML cells via inhibition of FOXO activity.

### Downregulation of TRIB1 by AS1842856 contributes to AML cell differentiation

To explore the molecular mechanism by which AS1842856 overcomes differentiation blockade in AML, we performed microarray analysis after treatment with AS1842856 for 6 h. Statistically significant differential expression between the vehicle-treated and AS1842856-treated groups was observed in 3361 genes, and 247 genes were downregulated, while 92 genes were upregulated by >2-fold in the AS1842856-treated group (Supplementary Fig. 1A). GSEA analysis revealed that AS1842856 inhibited pathways critical for leukemogenesis, such as MEIS1/HOXA9, NF–κB, and Evi1 (Supplementary Fig. 1B). These observations suggest that the inhibition of oncogenic pathways by the compound could be the cause of AML cell differentiation in various AML cell lines.

**Fig. 2:**
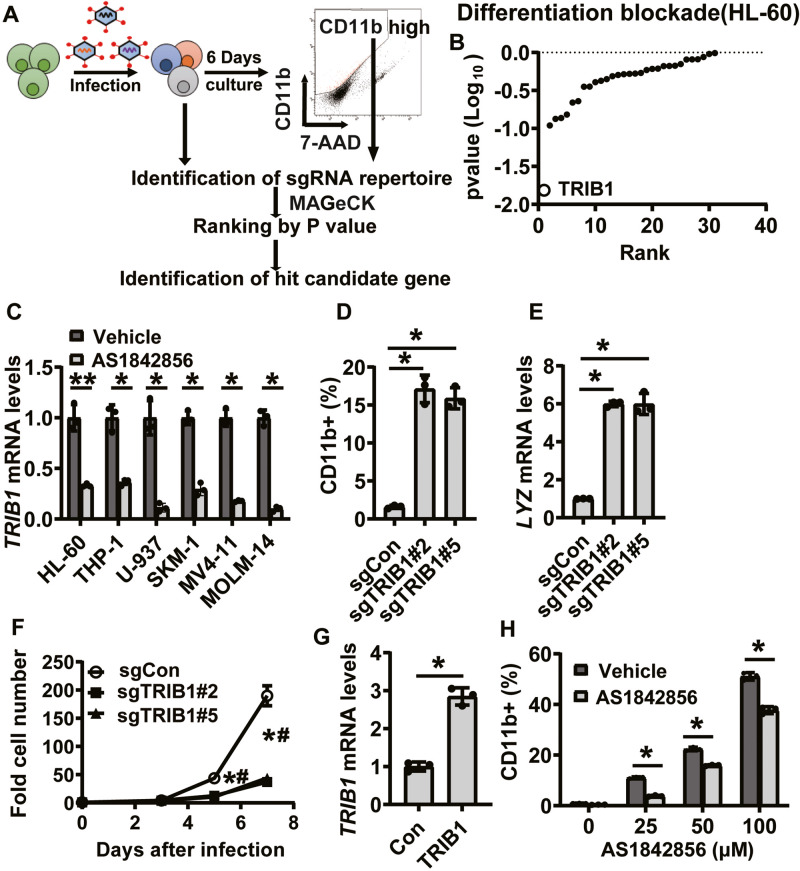
Downregulation of *TRIB1* by a FOXO1 inhibitor contributes to AML cell differentiation. **A** Schematic diagram of the strategy of CRISPR library screening for identification of critical molecules involved in AML cell differentiation by AS1842856. **B** Hit candidate genes of CRISPR screening for differentiation blockade. **C** RT-qPCR for quantification of *TRIB1* expression in AML cell lines treated with AS1842856 for 6 h. HL-60 and THIP-1 cells were treated with 100 nM AS1842856. U-937, SKM-1, MV4-11, MOLM-14 cells were treated with 4 μM AS1842856. *P < 0.01, **P < 0.05. Two-side Welch’s t-test. **D** FACS analysis of CD11b in HL-60 at 6 days after induction of sgRNA. Data are mean ± SD (*n* = 3). *P < 0.01. Two-side Welch’s t-test. **E** RT- qPCR for quantification of *LYZ* expression in HL-60 at 6 days after induction of sgRNA. Data are mean ± SD (*n* = 3). *P < 0.01. Two-side Welch’s t-test. **F** Cell proliferation of HL-60 after induction of sgRNA. Data are mean ± SD (*n* = 3). sgCon vs sgTRIB1#2; *P < 0.01, sgCon vs sgTRIB1#5; #P < 0.01. Two-side Welch’s t-test. **G** RT-qPCR for quantification of *TRIB1* expression in *TRIB1*-overexpressing HL-60 cells. *P < 0.01. Two-side Welch’s t-test. **H** FACS analysis of CD11b in HL-60 treated with AS1842856 for 3 days. Data are mean ± SD (*n* = 3). *P < 0.01. Two-side Welch’s t-test.

To identify critical molecules for AML cell differentiation by AS1842856, we performed CRISPR/Cas9 screening based on the selection of CD11b, a surface marker of myeloid differentiation. We constructed a custom CRISPR library containing sgRNAs targeting the top 30 protein-coding genes with the highest suppression ratios by AS1842856 and 1004 nontargeting sgRNAs as negative control. The sgRNA library was introduced into Cas9-expressing HL-60 cells via lentivirus (Fig. 2A). Following a 6-day culture, the CD11b-high cell fraction was isolated as a concentrated population of differentiated cells. Subsequently, the sgRNA repertoire of the sorted and unsorted samples was quantified, and next-generation sequencing was employed to identify enriched sgRNAs specifically in differentiated cells (Fig. 2A). Through ranking the target genes of the sgRNAs enriched within the CD11b-high population based on the p-values calculated by the MAGeCK algorithm, we identified *TRIB1* as a critical molecule in differentiation blockade (Fig. 2B). We also confirmed downregulation of *TRIB1* by AS1842856 in all tested AML cell lines (Fig. 2C). TRIB1 is a component of the COP1 ubiquitin ligase complex and plays a pivotal role in leukemogenesis and differentiation blockade by promoting proteolysis of CCAAT/enhancer binding proteins (C/EBPα and C/EBPβ), which are crucial inducers of differentiation in AML. Consistent with previous studies, knockout of *TRIB1* increased myeloid markers including CD11b and *LYZ* (Fig. 2D and E), resulting in inhibition of proliferation (Fig. 2F). Importantly, overexpression of *TRIB1* significantly restored the effects of AS1842856 on cell differentiation (Fig. 2G, H). These data revealed that *TRIB1* is a functional molecule for AML cell differentiation induced by AS1842856.

### Identification of cis-regulatory element in TRIB1 promoter to maintain the undifferentiated status of AML cells

**Fig. 3:**
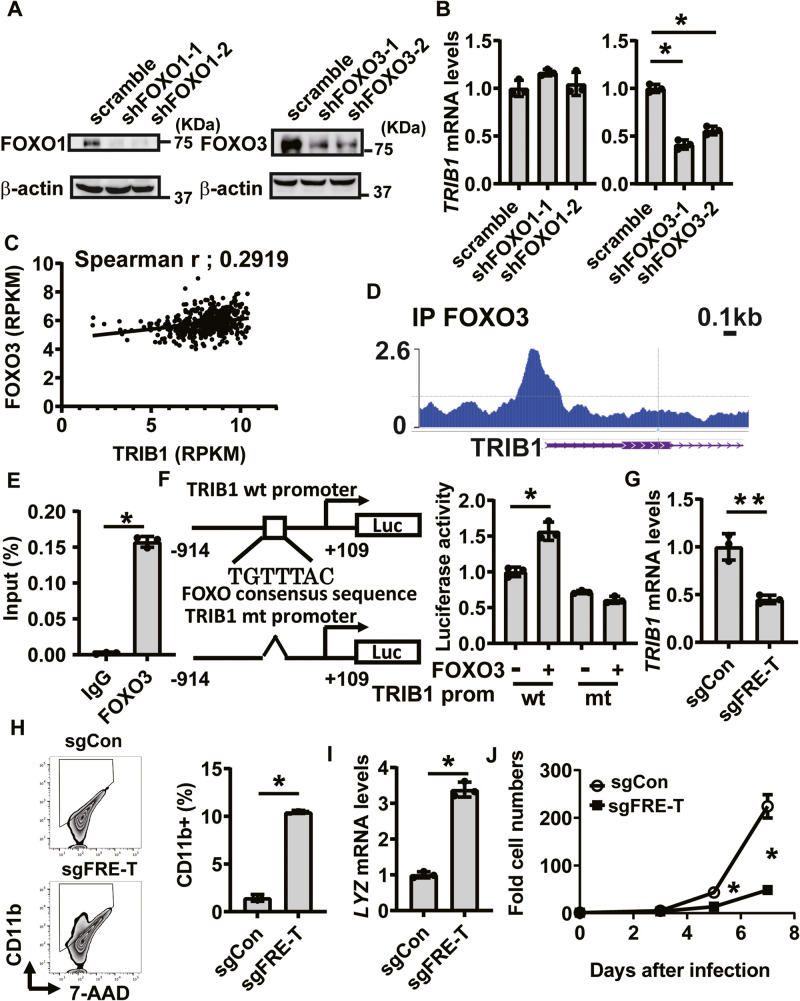
Identification of cis-regulatory element for *TRIB1* gene transcription to maintain the undifferentiated status of AML cells. **A** Western blotting of FOXO1 and FOXO3 protein levels in HL-60 at 3 days after induction of individual shRNA. **B** RT-qPCR for quantification of *TRIB1* expression in HL-60 at 3 or 4 days after induction of shRNA against FOXO1 or FOXO3, respectively. *P < 0.01. Two-side Welch’s t-test. **C** Gene expression levels of *FOXO3* and *TRIB1* in AML patient samples (*n* = 451). **D** ChIP-Seq data from Cistrome Data Browser for FOXO3 binding region in *TRIB1* promoter (CistromeDB: 74682). **E** ChIP qPCR for the interaction of FOXO3 with the *TRIB1* promoter in HL-60. Data are mean ± SD (*n* = 3). *P < 0.01. Two-side Welch’s t-test. **F** Luciferase assay for identification of the FOXO3 responsive element in the *TRIB1* promoter. Data are mean ± SD (*n* = 3). *P < 0.01. Two-side Welch’s t-test. **G** RT-qPCR for quantification of *TRIB1* expression in HL-60 at 2 days after the induction of sgRNA. Data are mean ± SD (*n* = 3). **P < 0.05. Two-side Welch’s t-test. **H** FACS analysis for CD11b in HL-60 at 6 days after induction of sgRNA. Data are mean ± SD. *P < 0.01. Two-side Welch’s t-test. **I** RT-qPCR for quantification of *LYZ* expression in HL-60 at 6 days after induction of sgRNA. Data are mean ± SD (*n* = 3). *P < 0.01. Two-side Welch’s t-test. **J** Cell proliferation of HL-60 after induction of sgRNA. Data are mean ± SD (*n* = 3). *P < 0.01. Two-side Welch’s t-test.

Next, we investigated how AS1842856 downregulated *TRIB1* gene expression. Knockdown of *FOXO3* significantly reduced *TRIB1* expression (Fig. 3A, B), while knockdown of *FOXO1* had no impact. Furthermore, FOXO3 expression was positively correlated with *TRIB1* expression in AML patient samples (Fig. 3C) [37]. A public database of chromatin immunoprecipitation (ChIP)-Seq and ChIP qPCR data revealed that FOXO3 are recruited to the *TRIB1* promoter (Fig. 3D, E). These data demonstrated that FOXO3 is a critical direct regulator of *TRIB1* expression. Because the FOXO binding region includes the FOXO binding consensus sequence (TGTTTAC), we examined whether this element is involved in the induction of *TRIB1* by FOXO3. Luciferase assay revealed that this element exhibits responsiveness to the constitutively active form of FOXO3 (Fig. 3F). Importantly, deletion of the FOXO responsive element in the *TRIB1* promoter by CRISPR/Cas9 significantly reduced *TRIB1* expression (Fig. 3G, Supplementary Fig. 2A). Thus, the FOXO responsive element plays a crucial role in *TRIB1* expression. We named this element FOXO-responsive element in the *TRIB1* promoter (FRE-T). Moreover, the deletion of FRE-T induced the expression of CD11b and *LYZ* associated with the inhibition of proliferation (Fig. 3H–J). These data demonstrated that a cis-regulatory element in the *TRIB1* promoter is essential to maintain the undifferentiated status of AML cells.

### Generation of pyrrole-imidazole polyamides (PIPs) that specifically target FRE for AML cell differentiation

**Fig. 4:**
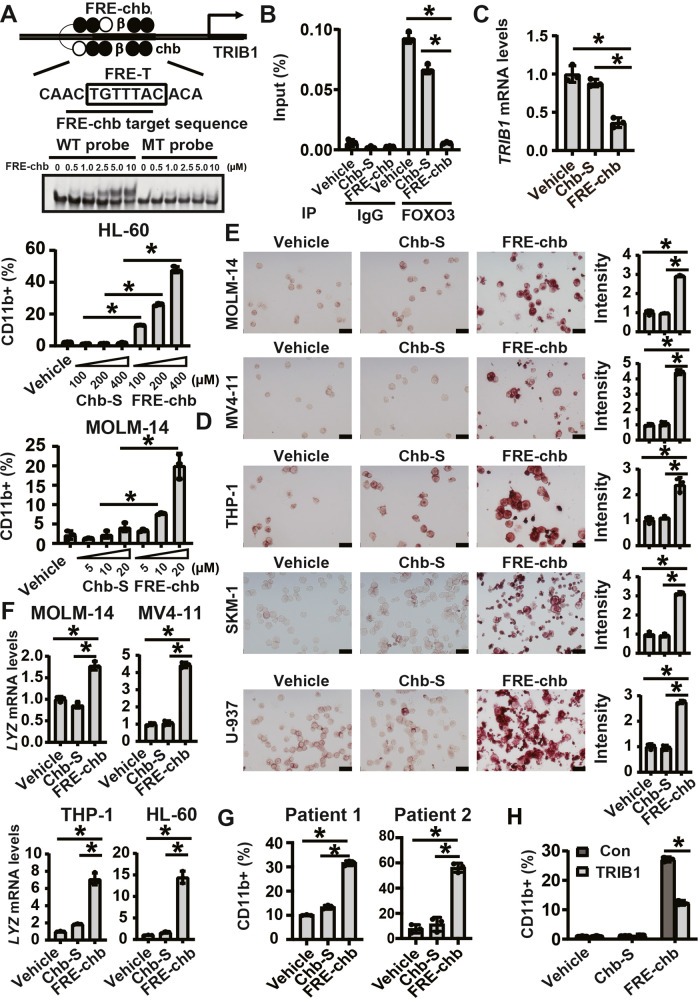
FRE-chb induces differentiation in a *TRIB1*-dependent manner. **A** EMSA for identification of the interaction between FRE-chb and TRE-T. **B** ChIP qPCR for quantification of the interaction between FOXO3 and the *TRIB1* promoter after 2 days of treatment with FRE-chb. Data are mean ± SD (*n* = 3). *P < 0.01. Two-side Welch’s t-test. **C** RT-qPCR for quantification of *TRIB1* expression in HL-60 after treatment with 400 nM FRE-chb for 4 days. Data are mean ± SD (*n* = 3). *P < 0.01. Two-side Welch’s t-test. **D** Quantification of CD11b-positive population in HL-60 and MOLM-14 after 4 days of treatment with FRE-chb. Data are mean ± SD (n = 3). *P < 0.01. Two-side Welch’s t-test. **E** NSE activity of individual cell lines treated with FRE-chb. MOLM-14 and MV4-11 cells were treated with 20 nM FRE-chb for 4 Days. THP-1 cells were treated with 200 nM FRE-chb for 3 Days. SKM-1 cells were treated with 100 nM FRE-chb for 5 Days. U-937 cells were treated with 400 nM FRE-chb for 4 Days. Data are mean ± SD (*n* = 3). *P < 0.01. Two-side Welch’s t-test. **F** RT-qPCR for quantification of *LYZ* in individual cell lines treated with FRE-chb. MOLM-14 cells were treated with 20 nM FRE-chb for 3 Days. MV4-11 cells were treated with 20 nM FRE-chb for 3 Days. THP-1 cells were treated with 200 nM FRE-chb for 4 Days. HL-60 cells were treated with 400 nM FRE-chb for 4 Days. Data are mean ± SD (*n* = 3). *P < 0.01. Two-side Welch’s t-test. **G** Quantification of CD11b-positive population in primary AML samples (Patient 1 and 2) after 4 or 7 days of treatment with FRE-chb, respectively. Data are mean ± SD (*n* = 3). *P < 0.01. Two-side Welch’s t-test. **H** Quantification of CD11b-positive population in HL-60 after 4 days of treatment with FRE-chb. Data are mean ± SD (*n* = 3). *P < 0.01. Two-side Welch’s t-test.

To develop a therapeutic methodology that involves the inhibition of FOXO function for the suppression of *TRIB1* expression, we attempted to inhibit the interaction between FOXOs and FRE-T (TGTTTAC) using PIPs conjugated to Chb (FRE-chb). To this end, the polyamide was designed to bind to ACTGTTTA, which includes a significant portion of the FRE-T, by the polyamide recognition rule [38] and to be alkylated at FRE-T (Fig. 4A, Supplementary Fig. 3A). The gamma turn portion was placed at the edge of the consensus sequence due to its strong AT selectivity [39]. Furthermore, since Py or Im cannot be consequent more than five, β-alanine, which can replace Py, was added in the middle [40].

First, we examined the binding specificity of FRE-chb to its targeting DNA sequence (ACTGTTTA) in TRIB1 promoter by electrophoretic mobility shift assays. The wild-type FRE-chb probe (WT probe) includes FRE-chb targeting sequence in the TRIB1 promoter and mutant type FRE-chb probe (MT probe) is mutated in its target sequence (Table S6). Because FRE-chb specifically binds to its target DNA sequence (Fig. 4A), we further examined the effects of FRE-chb. We confirmed that treatment with FRE-chb resulted in the inhibition of recruitment of FOXO3 to FRE-T in the genome and reduced *TRIB1* expression in AML cells (Fig. 4B, C). FRE-chb efficiently induced differentiation, as determined by CD11b, *LYZ*, and NSE activity in various AML cell lines (Fig. 4D–F). Moreover, FRE-chb increased the differentiation marker CD11b in primary AML patient samples (Fig. 4G). Importantly, the effects of FRE-chb on cell differentiation were restored upon overexpression of *TRIB1* (Fig. 4H). These data indicated that FRE-chb induces AML cell differentiation through the downregulation of *TRIB1*.

### Effectiveness of PIPs targeting FRE for AML therapy

**Fig. 5:**
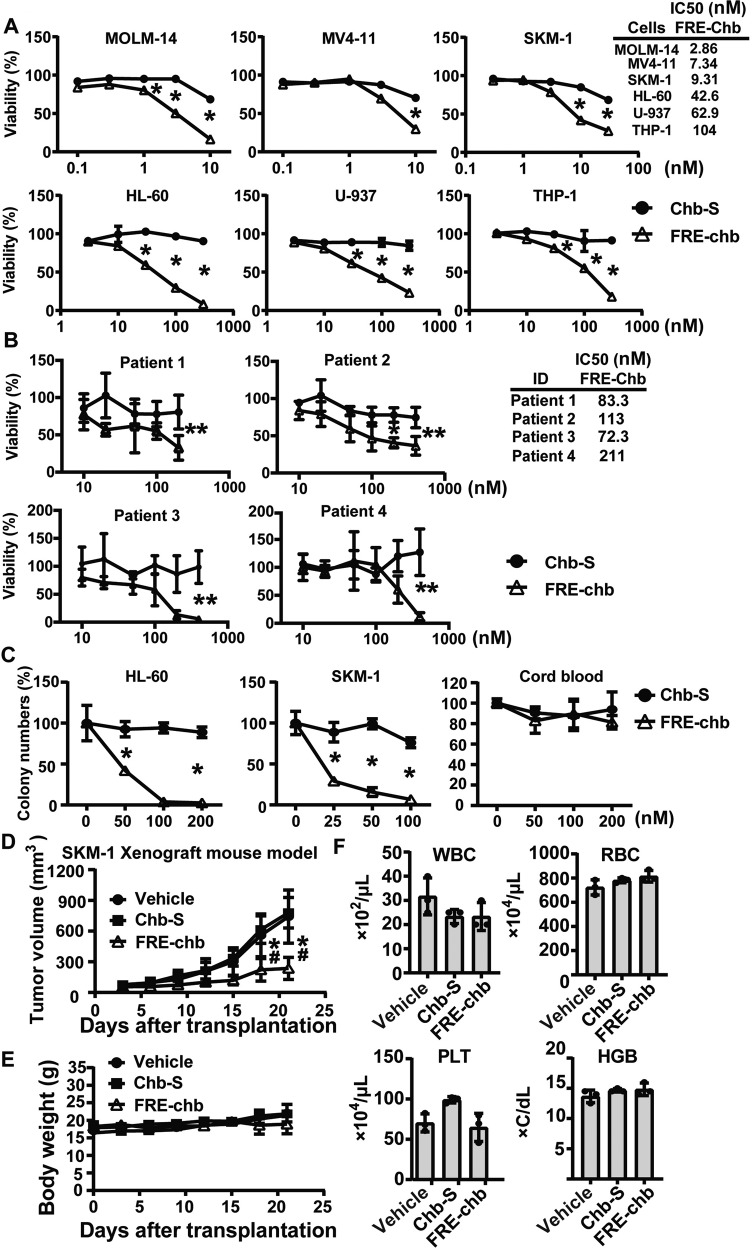
FRE-chb exhibits antitumor effects without remarkable side effects. **A** Quantification of IC50 values after treatment with individual reagents for 3 days. Data are mean ± SD (*n* = 3). Chb-S vs FRE-chb; *P < 0.01. Two-side Welch’s t-test. **B** Quantification of IC50 values after treatment with individual reagents for 7 days in AML primary samples. Data are mean ± SD (*n* = 3). Chb-S vs FRE-chb; *P < 0.01, **P < 0.05. Two-side Welch’s t-test. **C** Quantification of colony formation ability after treatment with individual reagents for 12 Days. Data are mean ± SD (*n* = 3). Chb-S vs FRE-chb; *P < 0.01. Two-side Welch’s t-test. **D** Quantification of antitumor efficiency of FRE-chb using SKM-1 xenograft mouse model. Data are mean ± SD (*n* = 6). Vehicle vs FRE-chb; *P < 0.01, Chb-S vs FRE-chb; #*P* < 0.01. Two-side Welch’s t-test. **E** Effect of FRE-chb on body weight. Data are mean ± SD (*n* = 3). **F** Effect of FRE-chb on blood component. Data are mean ± SD (*n* = 3).

Finally, to assess the antileukemia efficiency of FRE-chb, we evaluated its half-maximal inhibitory concentration (IC50) in six AML cell lines. FRE-chb significantly inhibited the proliferation of leukemia cells at concentrations in the nanomolar range compared with control PIP, Chb-S, which reportedly does not affect proliferation in AML cell lines (Fig. 5A). In four AML cell lines, the IC50 values of FRE-chb were lower than those of the FOXO inhibitor (AS1842856) (Figs. 1D and 5A). Notably, in MOLM-14, MV4-11, and SKM-1, the IC50 values of FRE-chb was less than 9-fold those of AS1842856 (Figs. 1D and 5A). These data indicate the superiority of FRE-chb to AS1842856 in terms of antileukemia efficacy. Importantly, FRE-chb reduces cell viability at nM levels in all AML patient samples (Fig. 5B) and these IC50 values are lower than those of AS1842856 after 7 days of treatment (Figs. 1E and 5B), underscoring the high potential of FRE-chb as an anticancer drug.

To investigate the therapeutic window of FRE-chb, we compared the colony formation efficiency between AML cell lines and human hematopoietic stem cells (HSCs; CD34+ cells) from cord blood treated with FRE-chb. The sensitivity of AML cell lines to FRE-chb was much higher than that of HSCs, indicating the therapeutic advantage of FRE-chb in AML (Fig. 5C). To examine the effectiveness of FRE-chb in vivo, we evaluated its antitumor effects using a SKM-1 xenograft model. Administration of FRE-chb significantly suppressed progression of tumors (Fig. 5D) without remarkable adverse effects (Fig. 5E, F). Taken together, these results indicate that FRE-chb is a promising therapeutic strategy for the treatment of AML.

## Discussion

Although the FOXO family of transcription factors represents a promising target for differentiation therapy in AML, the efficacy of FOXO inhibitors in vivo is limited. In this study, we developed a novel type of FOXO inhibitor targeting cis-regulatory elements of the FOXO family (FRE) using a PIP conjugated to Chb (FRE-chb). FRE-chb induced differentiation in various mutated AML cell lines. Mechanistically, FRE-chb induced differentiation by inhibiting the induction of *TRIB1* expression by FOXO3. Therefore, inhibiting FOXO signaling using PIPs is a promising therapeutic strategy for AML.

A critical finding of our research was that the FOXO inhibitor (AS18428569) induced differentiation in various AML cell lines. Although Lin et al. showed that AS1842856 inhibits the proliferation of AML1-ETO-expressing AML cell lines, the efficiency of the compound against other mutation statuses of AML and the mechanistic action has not been elucidated [15]. We found that AS1842856 exerts its antiproliferative effects by inducing differentiation in various mutated AML cell lines. AS1842856 is known to suppress the transcriptional activity of FOXOs, especially FOXO1. Additionally, knockdown of *FOXO3* induces differentiation in human AML cell lines harboring different types of mutations, and FOXO1 overexpression is sufficient to induce differentiation blockade and a preleukemic state in HSPCs. Considering these observations, FOXO1 and FOXO3 could be crucial regulators of differentiation blockade and represent promising drug targets for differentiation therapy.

Although FOXO downstream genes have been characterized through RNA-seq and ChIP-seq analyses, the mechanism of differentiation blockade by FOXOs remains unclear because of the uncertainty of their functional target genes [14, 15]. Through analysis of the pharmacological effects of AS1842856, we identified *TRIB1*, a component of the COP1 ubiquitin ligase complex, as a functional molecule involved in compound-induced AML cell differentiation. Substrates of the COP1 ubiquitin ligase complex include transcription factor C/EBPα, whose overexpression induces differentiation in AML. Inactivating mutations of C/EBPα are detected in 10–15% of AML cases and promote leukemia in mice, highlighting the importance of C/EBPα in differentiation blockade [41]. Furthermore, overexpression of *TRIB1* reduced C/EBPα protein levels, resulting in progression of leukemia in murine models [42, 43]. These findings indicate that *TRIB1* is a crucial oncogene regulating C/EBPα and differentiation status in AML. Importantly, we identified the FOXO-responsive element in the *TRIB1* promoter (FRE-T) and found that transcriptional activation of *TRIB1* though FRE-T plays a critical role in AML differentiation blockade. These data support that the FOXO–*TRIB1* axis is an important pathway maintaining the undifferentiated status of AML.

However, the partial restoration of AS1842856-induced differentiation by overexpression of *TRIB1* suggests that other FOXO targets also contribute to the maintenance of the undifferentiated state in AML. The FOXO downstream genes include *MYC*, a crucial molecule of leukemogenesis and differentiation blockade in AML. Smita et al. revealed that FOXOs are important molecules for maintaining *MYC* expression in breast cancer [44]. Consistent with this study, publicly available databases indicate that FOXO3 associates with the 5′UTR of *MYC*, and knockdown of *FOXO3* reduces *MYC* expression in AML cell lines (Supplementary Fig. 4A and B). Importantly, knockout of *MYC* clearly induces differentiation in HL-60 cells (Supplementary Fig. 4C). Thus, the FOXO–*MYC* axis could be a critical node for differentiation blockade in AML. In contrast, FOXOs repress *MYC* expression through induction of *miR-34c* expression [45]. Thus, the regulation of *MYC* expression by FOXOs varies according to cellular circumstances and environments, underscoring the high therapeutic efficacy of FOXO inhibitors in AML without remarkable adverse effects on various organs.

Transcription factors are generally regarded as undruggable targets. In this study, we developed a novel type of FOXO inhibitor that targets common cis-regulatory elements of FOXOs, including FRE-T, using PIPs conjugated to chlorambucil (FRE-chb). We clearly demonstrated that FRE-chb efficiently impedes the FOXO–*TRIB1* axis, thereby inducing differentiation in various AML cell lines. Thus, we successfully developed a new differentiation agent using PIPs targeting FOXO target genes. Importantly, FRE-chb specifically inhibited AML colony formation, in contrast to cord blood, and exhibited antitumor effects without remarkable adverse effects in vivo. These data suggest the considerable therapeutic potential of FRE-chb in the treatment of AML. One potential explanation for its selective therapeutic efficacy and induction of differentiation may be the specific dependency of FOXO1 and FOXO3 in AML, as evidenced by their elevated expression levels in these cells (Supplementary Fig. 5A). Additionally, given that certain PIPs exhibit a tendency to accumulate in tumors, it is possible that FRE-chb may also accumulate in tumors, thereby conferring selective toxicity to tumors [21]. On the basis of these findings, we propose the repression of FOXO-related gene expression via PIPs as a novel therapeutic strategy for AML cell differentiation. To improve the therapeutic efficiency of FOXO inhibitors utilizing PIPs, further research identifying functional FOXO downstream genes and cis-regulatory elements is necessary.

### Supplementary information

Supplementary Fig. 1-5

Table S1-S6

supplemental material

Checklist

Original Data File

Consent for Authorship Change

## Data Availability

The Microarray data has been deposited in the Gene Expression Omni-bus (GEO) database, under number GSE227114. All data and additional information about the experiments reported in this paper will be shared upon reasonable request.
