# Supplementary material for: Targeting cis-regulatory elements of FOXO family is a novel therapeutic strategy for induction of leukemia cell differentiation
Source: Cell Death Dis. 2023 Sep 29;14(9):642. doi: 10.1038/s41419-023-06168-2 (PMC10541907; doi:10.1038/s41419-023-06168-2)
Supplement: Supplementary file 1 — Supplementary Fig. 1-5 [file 41419_2023_6168_MOESM1_ESM.docx]

**Supplementary Figures**


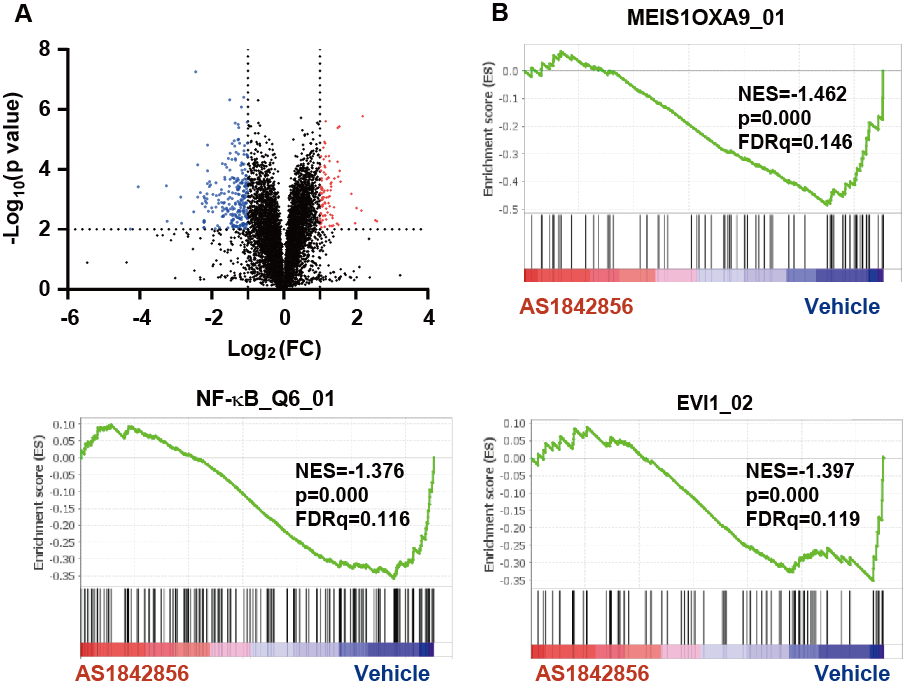


**Supplementary Fig. 1**

AS1842856 inhibited critical pathways for leukemogenesis. (A) Volcano plot for significantly altered gene expressions by AS1842856 treatment. (B) GSEA enrichment curves of each data set.


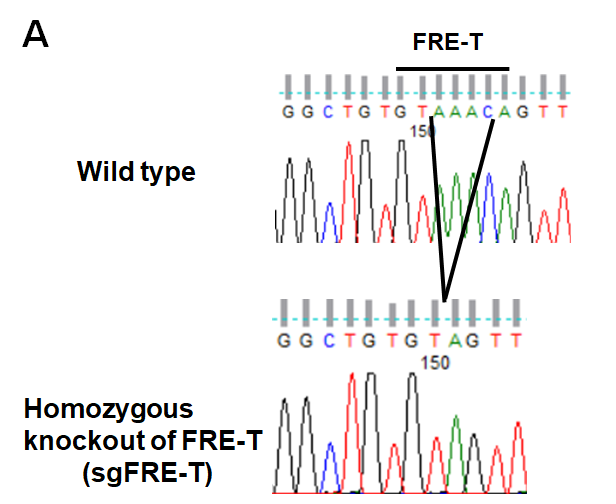


**Supplementary Fig. 2**

CRISPR/Cas9 system knockout of FRE-T. (A) Confirmation of CRISPR/Cas9-mediated depletion of FRE-T sequence.


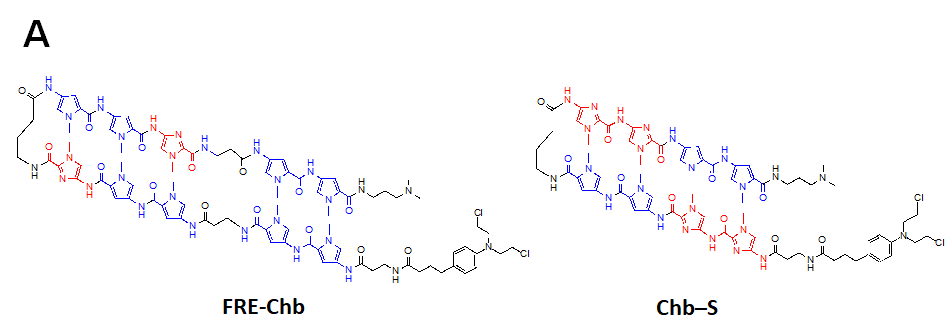


**Supplementary Fig. 3**

Chemical structures of FRE-chb and Chb-S.


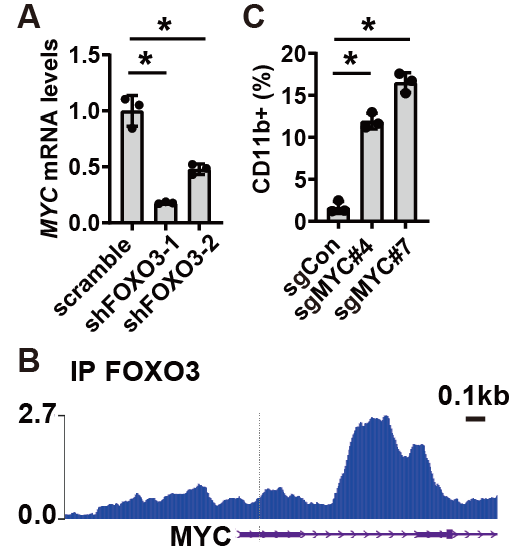


**Supplementary Fig. 4**

FOXOs regulated *MYC* expression in HL-60. (A) RT-PCR analysis for quantification of *MYC* expression in HL-60 after knockdown of *FOXO3*. Data are mean ± SD (n = 3). *P < 0.01. (B) ChIP-Seq data from Cistrome Data Browser for the FOXO3 binding region in the *MYC* promoter (CistromeDB: 74682). (C) FACS analysis of CD11b in HL-60 at 3 days after knockout of *MYC*. Data are mean ± SD (n = 3). *P < 0.01.


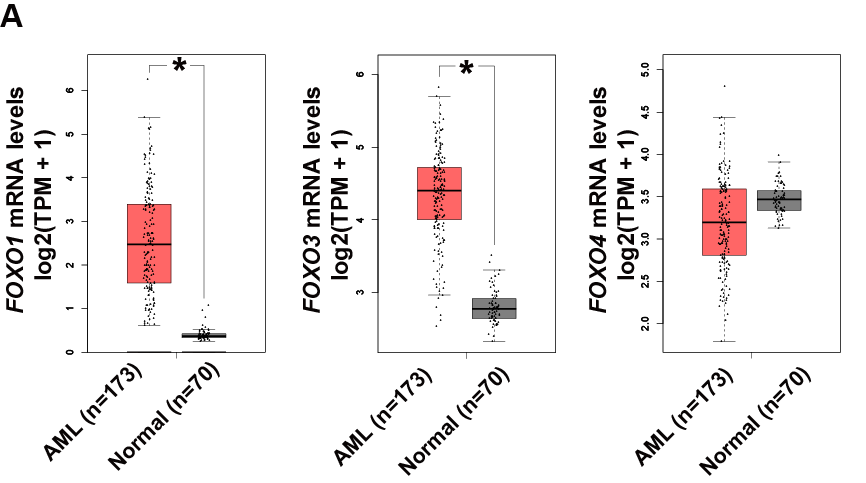


**Supplementary Fig. 5**

Upregulation of FOXOs in AML patients. (A) Data from GEPIA for gene expression of *FOXO1*, *FOXO3*, and *FOXO4* in AML patients and healthy donors. *P < 0.01.
