# Supplementary material for: Targeting cis-regulatory elements of FOXO family is a novel therapeutic strategy for induction of leukemia cell differentiation
Source: Cell Death Dis. 2023 Sep 29;14(9):642. doi: 10.1038/s41419-023-06168-2 (PMC10541907; doi:10.1038/s41419-023-06168-2)
Supplement: Supplementary file 3 — supplemental material [file 41419_2023_6168_MOESM3_ESM.docx]

**Supplementary Methods**

**Cell culture**

293T cells were cultured in DMEM supplemented with 10% fetal bovine serum (FBS), penicillin (100 units/ml; Fujifilm Wako Chemical, Osaka, Japan), and streptomycin (100 μg/ml; Fujifilm Wako Chemical). THP-1, HL-60, MOLM-14, MV4-11, U-937, and SKM-1 cells were cultured in RPMI-1640 supplemented with 10% FBS, penicillin, and streptomycin. 293T, HL-60, THP1, and human umbilical cord blood were obtained from RIKEN BRC (Ibaraki, Japan). U-937, SKM-1, and MOLM-14 were purchased from JCRB Cell Bank (Osaka, Japan). MV4-11 cells were obtained from the American Type Culture Collection (Manassas, Virginia, USA). Cas9-expressing HL-60 cells were developed by induction of pUBCP-FLAG-Cas9-P2A-Puro or Lenti-CRISPRv2 with lentivirus. TRIB1-overexpresssing HL-60 were developed by induction of pLJM1 TRIB1.

**Microarray analysis**

THP-1 cells were treated with 0.1 μM AS1842856 for 6 h and RNA was extracted using an RNeasy Mini Kit (QIAGEN) according to the manufacturer's instructions. The results of the microarray were analyzed by GeneSpringGX (Agilent). Gene set enrichment analysis (GSEA) was performed using the Broad Institute GSEA softwarehttps://www.broadinstitute.org/gsea/.

**Analysis of patient-derived AML samples**

After purification of mononuclear cells by density‐gradient centrifugation using Ficoll‐Hypaque (Sigma‐Aldrich), primary AML cells were cultured in the MethoCult H4534 Classic without EPO (Stem Cell Technologies), and were exposed to AS1842856 or FRE-chb for 4 or 7 or 12 days. APC-conjugated Anti-human CD11b antibody (BD Biosciences) were used to discern the CD11b+ population in patient-derived AML samples, and cell viability using was measured using CellTiter-Glo® Luminescent Cell Viability Assay (Promega) according to the manufacturer's instructions. Information regarding AML primary samples were shown in Table S3.

**Cell viability and proliferation**

To determine IC50s for the AML cell lines, cells were cultured with AS1842856 or FRE-chb for 6 days and 3 days, respectively, and cell viability was measured using the Cell Counting Kit 8 (Dojindo) according to the manufacturer's instructions. Absorbance was measured using an Infinite Pro 200 Reader (Tecan). Cell counting for the growth curve was performed using a TC20 Cell Counter (Bio-Rad). To quantify the cell viability of primary AML samples, cells were cells were cultured with AS1842856 or FRE-chb for 7 or 12 days and cell viability was measured using the CellTiter-Glo® Luminescent Cell Viability Assay (Promega) according to the manufacturer's instructions. Luminescence were measured using an Infinite Pro 200 Reader (Tecan).

**Colony forming assay**

CD34+ Lin− cells were isolated from human cord blood using a Diamond CD34 Isolation Kit human (Miltenyi Biotec) according to the manufacturer's instruction. The isolated CD34+ Lin- cells were seeded at a density of 500 cells/mL in 1 mL MethoCult H4034 Optimum (StemCell Technologies) supplemented with 100 ng/ml FLT3 ligand and 100 ng/ml human recombinant TPO. After 12 days of culture, the number of colonies was quantified using an All-in-One Fluorescence Microscope BZ-X800 (KEYENCE). SKM-1 and HL-60 cells were seeded at a density of 500 cells/mL and 300 cells/mL in 1 mL1.5% methylcellulose media supplemented with 20% FBS and penicillin/streptomycin. After 12 days of culture, the number of colonies was quantified.

**Differentiation markers**

For flow cytometry analysis, cells were collected and incubated with APC-conjugated anti-human CD11b antibody (BD Biosciences, Cat#550019) for 30 mins on ice. The cells were washed with 2% FBS-supplemented PBS (F-PBS) and resuspended in F-PBS with 7-AAD (BioLegend). The CD11b-positive population was quantified using FACS Lyric™ (BD Biosciences). For non-specific esterase (NSE) analysis, cells were plated on a glass slide by centrifugation and then stained for NSE activity using a staining kit (Muto Pure Chemical, Tokyo, Japan) in accordance with the manufacturer's instructions. The quantification of the intensity of NSE activity was performed using ImageJ software.

**Western blot**

Immunoblot analysis was performed as described previously [1]. The primary antibodies used were anti-FOXO1 (Cell Signaling Technology, C29H4; 1:1000 with Canget Signal (TOYOBO)), anti-FOXO3 (Cell Signaling Technology, 75D8; 1:1000 with Canget Signal (TOYOBO)), and anti-β-actin (Sigma–Aldrich, A5441; 1:2000 with TBST). Isotype-matched secondary antibodies conjugated with horseradish peroxidase (Cytiva, Cat# NA9310-1ML, Cat# NA9340-1ML) were used and Proteins were detected using ImmunoStar® LD (FUJIFILM Wako) and LAS4000 (GE Healthcare).

**RT-qPCR**

RNA was extracted using an RNeasy Mini Kit (QIAGEN) according to the manufacturer’s instructions. Complementary DNA (cDNA) was synthesized from the extracted RNA using an Advantage® RT-for-PCR Kit (Clontech Laboratories, Inc.). Quantitative real-time PCR (qRT-PCR) was performed using the Mx3000P qPCR System (Agilent). The gene expression data were normalized to GAPDH as an internal control. Primer sequences for qRT-PCR are listed in Table S4.

**ChIP qPCR**

Cells were cross-linked with 0.5% formaldehyde for 10 minutes, and the reaction was quenched with 0.125 M glycine for 5 minutes. The cells were washed with PBS and lysed with NP-40 buffer (10 mM Tris-HCl (pH 8.0), 10 mM NaCl, and 0.5% NP-40) on ice for 5 minutes. Following centrifugation, the pellet was resuspended in 100 μL dissolution buffer (50 mM Tris-HCl (pH 8.0), 176 mM NaCl, and 1.1% sodium deoxycholate) and further mixed with 400 μL ChIP diluent buffer (10 mM Tris-HCl (pH 8.0), 1% SDS, and 10 mM EDTA (pH 8.0)). After sonication and centrifugation, the soluble chromatin was immunoprecipitated with a complex of magnetic beads and a FOXO3 antibody (ab12162; Abcam) at 4°C. The immunoprecipitated complex was subsequently washed once with 150 mM RIPA buffer (50 mM Tris-HCl (pH 8.0), 150 mM NaCl, 1 mM EDTA (pH 8.0), 0.1% SDS, 1% Triton X-100, and 0.1% sodium deoxycholate) and 500 mM RIPA buffer (50 mM Tris-HCl (pH 8.0), 500 mM NaCl, 1 mM EDTA (pH 8.0), 0.1% SDS, 1% Triton X-100, and 0.1% sodium deoxycholate), followed by two washes with TE buffer. The bound DNA was subsequently eluted with 200 μL elution buffer (10 mM Tris-HCl (pH 8.0), 300 mM NaCl, 5 mM EDTA (pH 8.0), and 0.5% SDS) and reverse-cross-linked at 65°C. The DNA samples were then incubated sequentially with 1 μg RNase and 100 μg Proteinase K, and purified with phenol/chloroform. ChIP-qPCR analysis was performed using an Mx3000P qPCR system (Agilent). Primer sequences for ChIP-qPCR are listed in Table S5.

**Electrophoretic mobility shift assay**

Wild type FRE-chb probe (WT probe) and mutant type FRE-chb probe (MT probe), in which the target sequence of FRE-chb (ACTGTTTA) was substituted for CAGAGGGA, were prepared by annealing oligonucleotides listed in Table S6. FRE-chb was incubated with the DNA duplex (12 µM) in reaction buffer (13.3 mM NaCl, 13.3 mM KCl) for 1 hour. After loading dye was added, the mixtures were loaded onto 20% polyacrylamide gels and electrophoresis was performed. Following staining DNA probes with ethidium bromide, the signals were detected using the FUSION FX7 EDGE Imaging System (Witec AG).

**Synthesis of FRE-chb**

Reagents and solvents were purchased from standard suppliers and used without further purification. Automated polyamide synthesis was performed on a PSSM-8 system (Shimadzu). HPLC analysis of each synthesized compound was performed on a Jasco Engineering PU-2089 plus series system using a Chemcobond 5-ODS-H 4.6 mm × 150 mm column (Chemco Plus Scientific) in 0.1% TFA in water with acetonitrile as the eluent at a flow rate of 1.0 mL/min and a linear gradient elution of 0%–100% acetonitrile in 40 min with detection at 254 nm. Collected fractions were analyzed by MALDI-TOF-MS Microflex-KS II (Bruker).

The solid-phase synthesis of each PIP was performed on a PSSM-8 system (Shimadzu), as described previously [2]. The building blocks used in this study were FmocHN-Py-CO2H, FmocHN-Im-CO2H, FmocHN-β-alanine-CO2H, and FmocHN-γ-aminobutyric acid. Each of them was introduced sequentially to 86 mg FmocHN-Py-oxime resin (0.44 mmol/g). Following cleavage reaction with 1000 μL N, N-dimethyl-1,3-propanediamine at 55°C for 3 hours, filtration and Et2O precipitation generated 52.2 mg of H2N-β-PyPyβPyPyIm-γ-PyPyImβPyPy-Dp. The crude polyamide (52.2 mg) dissolved in 522 μL dimethylformamide was mixed with chlorambucil (Chb, 19 mg, 2 equiv.), benzotriazole-1-yl-oxy-tris-pyrrolidino-phosphonium hexafluorophosphate (PyBOP, 67 mg, 4 equiv.), and N, N-diisopropylethylamine (DIEA, 65 μL ,12 equiv.) and stirred at room temperature for 2 hours. After Et2O precipitation, Chb-HN-β-PyPyβPyPyIm-γ-PyPyImβPyPy-Dp (FRE-chb) was obtained. This crude sample was dissolved in DMF and purified by HPLC to obtain pure FRE-chb as an off-white powder (24.1 mg, analytical HPLC: tR = 21.6 min. MALDI-TOF MS: m/z calculated for C90H112Cl2N29O15+[M+H]+1908.82, found; 1910.41).

**Luciferase assay**

The reporter and effector plasmids were transfected using PEI max (Polysciences, Inc.GE Healthcare). Luciferase assays proceeded as previously described [3]. To control for transfection efficiency, luciferase reporter plasmids including *TRIB1* promoter were co-transfected with an EF1 core promoter-driven *Renilla* luciferase construct as an internal control, and luciferase activity was normalized to *Renilla* activity.

1. Iwanaga R, Ohtani K, Hayashi T, Nakamura M. Molecular mechanism of cell cycle progression induced by the oncogene product Tax of human T-cell leukemia virus type I. Oncogene. 2001; 20: 2055-2067.

2. Asamitsu S, Kawamoto Y, Hashiya F, Hashiya K, Yamamoto M, Kizaki S*, et al.* Sequence-specific DNA alkylation and transcriptional inhibition by long-chain hairpin pyrrole-imidazole polyamide-chlorambucil conjugates targeting CAG/CTG trinucleotide repeats. Bioorg Med Chem. 2014; 22: 4646-4657.

3. Kurayoshi K, Shiromoto A, Ozono E, Iwanaga R, Bradford AP, Araki K*, et al.* Ectopic expression of the CDK inhibitor p21(Cip1) enhances deregulated E2F activity and increases cancer cell-specific cytotoxic gene expression mediated by the ARF tumor suppressor promoter. Biochem Biophys Res Commun. 2017; 483: 107-114.
