# Supplementary figures and images for: Targeting cis-regulatory elements of FOXO family is a novel therapeutic strategy for induction of leukemia cell differentiation
Source: Cell Death Dis. 2023 Sep 29;14(9):642. doi: 10.1038/s41419-023-06168-2 (PMC10541907; doi:10.1038/s41419-023-06168-2)

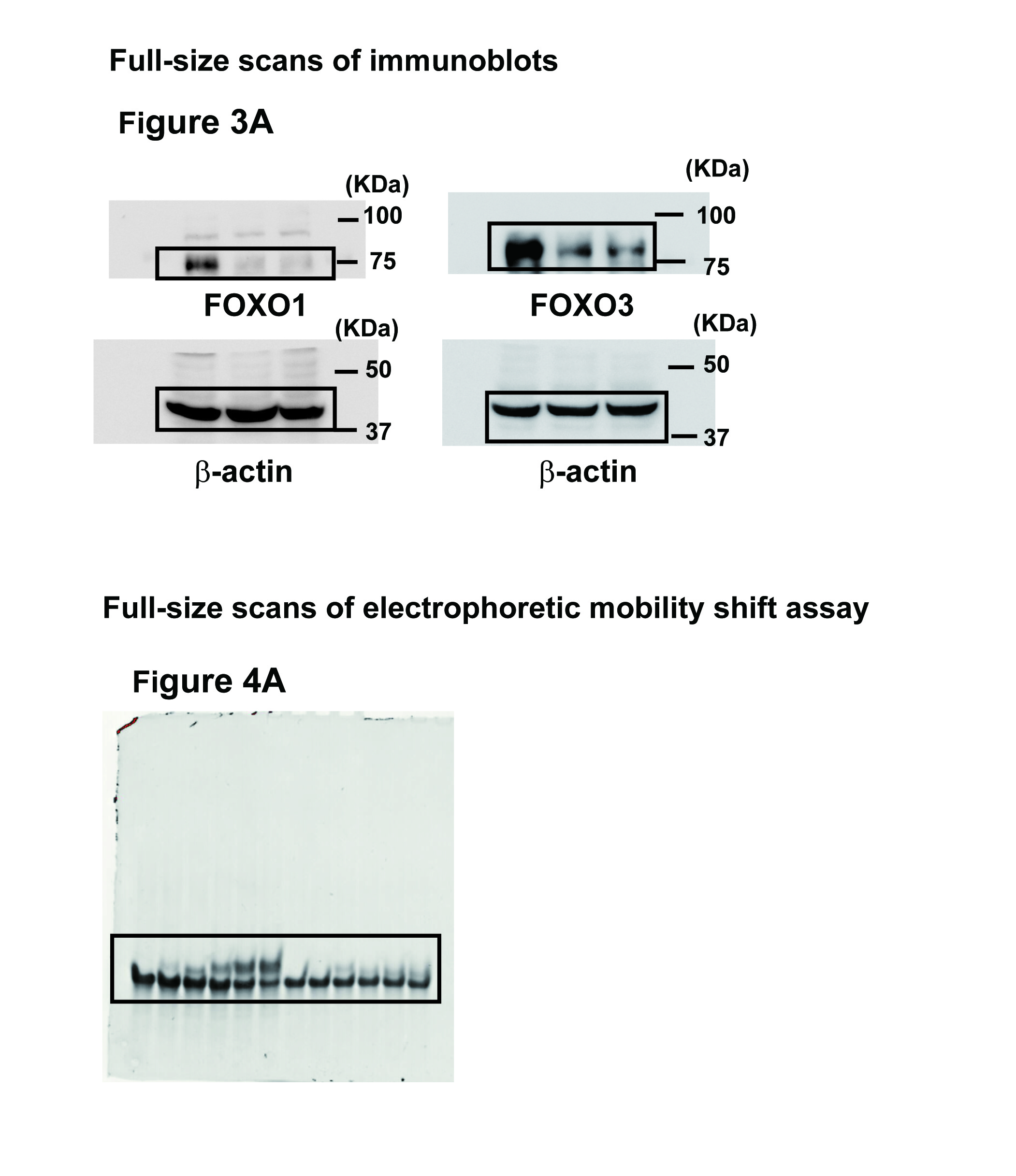

Supplement: Supplementary file 5 — Original Data File [file 41419_2023_6168_MOESM5_ESM.jpg]
