# Supplementary material for: Targeting cis-regulatory elements of FOXO family is a novel therapeutic strategy for induction of leukemia cell differentiation
Source: Cell Death Dis. 2023 Sep 29;14(9):642. doi: 10.1038/s41419-023-06168-2 (PMC10541907; doi:10.1038/s41419-023-06168-2)
Supplement: Supplementary file 6 — Consent for Authorship Change [file 41419_2023_6168_MOESM6_ESM.pdf]

Subject: Re: Change of author list
From: 布施香子 <kyo-fuse@med.niigata-u.ac.jp>
Date: 2023/09/18 20:42
To: Atsushi Hirao <ahirao@staff.kanazawa-u.ac.jp>

——————————
Dear Dr. Hirao,

I approve the change of the author list.
Best,

KYOKO FUSE

--- "平尾 敦　先生" <ahirao@staff.kanazawa-u.ac.jp> wrote:

> Dear Co-Authors,
>
> I would like to extend our heartfelt gratitude for your invaluable contributions to the preparation of our paper titled, “Targeting cis-regulatory elements of FOXO family is a novel therapeutic strategy for induction of leukemia cell differentiation” for publication in Cell Death & Disease. In the process of revising the manuscript, we have included Dr. Hitoshi Kiyoi and Dr. Yuichi Ishikawa from Nagoya University as co-authors, in addition to the original authors listed at the time of submission. Both Dr. Kiyoi and Dr. Ishikawa played pivotal roles in the analysis of cells from AML patients, significantly enhancing the quality of our research.
>
> We kindly request your concurrence with the inclusion of these esteemed colleagues as co-authors. Your approval would be greatly appreciated.If you concur with my suggestion, kindly respond with a straightforward statement, such as "I approve the change of the author list."
>
> Thank you sincerely for your cooperation.
>
> Warm regards,
>
> Atsushi Hirao
>
> --
> -----------------------

> Atsushi Hirao, M.D. Ph.D.
>
> Professor
> Division of Molecular Genetics
> Cancer Research Institute
> WPI Nano Life Science Institute(WPI-Nano LSI)
> Kanazawa University
>
> Kakuma-machi, Kanazawa, Ishikawa, Japan 920-1192
> TEL +81-76-264-6755
> FAX+81-76-234-4508
> e-mail ahirao@staff.kanazawa-u.ac.jp

Subject: Re: Change of author list
From: MAKIKO HORIKE <mhorike@staff.kanazawa-u.ac.jp>
Date: 2023/09/18 20:02
To: Atsushi Hirao <ahirao@staff.kanazawa-u.ac.jp>

Dear Dr. Hirao,

I approve the change of the author list.
Best,

Makiko Meguro-Horike

Makiko Meguro-Horike, PhD
Division of Integrated Omics research
Research Center for Experimental Modeling of Human Disease
Kanazawa University
13-1 Takara-machi
Kanazawa, 920-0934, Japan
Phone: +81(76)265-2775
Fax: +81(76)234-4537

2023年9月18日(月) 16:10 Atsushi Hirao <ahirao@staff.kanazawa-u.ac.jp>:

> Dear Co-Authors,
>
> I would like to extend our heartfelt gratitude for your invaluable contributions to the preparation of our paper titled, “Targeting cis-regulatory elements of FOXO family is a novel therapeutic strategy for induction of leukemia cell differentiation” for publication in Cell Death & Disease. In the process of revising the manuscript, we have included Dr. Hitoshi Kiyoi and Dr. Yuichi Ishikawa from Nagoya University as co-authors, in addition to the original authors listed at the time of submission. Both Dr. Kiyoi and Dr. Ishikawa played pivotal roles in the analysis of cells from AML patients, significantly enhancing the quality of our research.
>
> We kindly request your concurrence with the inclusion of these esteemed colleagues as co-authors. Your approval would be greatly appreciated.If you concur with my suggestion, kindly respond with a straightforward statement, such as "I approve the change of the author list."
>
> Thank you sincerely for your cooperation.
>
> Warm regards,

Atsushi Hirao

--

Atsushi Hirao, M.D. Ph.D.

Professor
Division of Molecular Genetics
Cancer Research Institute
WPI Nano Life Science Institute(WPI-Nano LSI)
Kanazawa University

Kakuma-machi, Kanazawa, Ishikawa, Japan 920-1192
TEL +81-76-264-6755
FAX+81-76-234-4508
e-mail ahirao@staff.kanazawa-u.ac.jp

Subject: Re: Change of author list
From: Shin-ichi Horike <sihorike@staff.kanazawa-u.ac.jp>
Date: 2023/09/18 20:27
To: Atsushi Hirao <ahirao@staff.kanazawa-u.ac.jp>

Dear Dr. Hirao,

I approve the change of the author list.
Best,

Shin-ichi Horike
--
Shin-ichi Horike, PhD
Division of Integrated Omics research
Research Center for Experimental Modeling of Human Disease
Kanazawa University
13-1 Takara-machi
Kanazawa, 920-0934, Japan
Phone: +81(76)265-2775
Fax: +81(76)234-4537
URL:http://chromosome.w3.kanazawa-u.ac.jp/horike-e/index.html

2023年9月18日(月) 16:10 Atsushi Hirao <ahirao@staff.kanazawa-u.ac.jp>:

> Dear Co-Authors,
>
> I would like to extend our heartfelt gratitude for your invaluable contributions to the preparation of our paper titled, “Targeting cis-regulatory elements of FOXO family is a novel therapeutic strategy for induction of leukemia cell differentiation” for publication in Cell Death & Disease. In the process of revising the manuscript, we have included Dr. Hitoshi Kiyoi and Dr. Yuichi Ishikawa from Nagoya University as co-authors, in addition to the original authors listed at the time of submission. Both Dr. Kiyoi and Dr. Ishikawa played pivotal roles in the analysis of cells from AML patients, significantly enhancing the quality of our research.
>
> We kindly request your concurrence with the inclusion of these esteemed colleagues as co-authors. Your approval would be greatly appreciated.If you concur with my suggestion, kindly respond with a straightforward statement, such as "I approve the change of the author list."
>
> Thank you sincerely for your cooperation.
>
> Warm regards,

Atsushi Hirao

--

Atsushi Hirao, M.D. Ph.D.

Professor
Division of Molecular Genetics
Cancer Research Institute
WPI Nano Life Science Institute(WPI-Nano LSI)
Kanazawa University

Kakuma-machi, Kanazawa, Ishikawa, Japan 920-1192
TEL +81-76-264-6755
FAX+81-76-234-4508
e-mail ahirao@staff.kanazawa-u.ac.jp

Subject: Re: Change of author list
From: Yuichi Ishikawa <yishikaw@med.nagoya-u.ac.jp>
Date: 2023/09/18 20:48
To: 平尾 敦先生 <ahirao@staff.kanazawa-u.ac.jp>
CC: Yuichi Ishikawa <yishikaw@med.nagoya-u.ac.jp>

Dear Dr. Hirao,

I approve the change of the author list.

Best regards,

Yuichi Ishikawa MD. PhD.

Department Hematology and Oncology
Nagoya University Graduate School of Medicine
65 Tsurumai-cho, Showa-ku, Nagoya, Japan 466-8550
TEL: +1-52-744-2139 FAX: +1-52-744-2157
E-mail: yishikaw@med.nagoya-u.ac.jp

> Dear Co-Authors,
>
> I would like to extend our heartfelt gratitude for your invaluable contributions to the preparation of our paper titled, "Targeting cis-regulatory elements of FOXO family is a novel therapeutic strategy for induction of leukemia cell differentiation" for publication in Cell Death & Disease. In the process of revising the manuscript, we have included Dr. Hitoshi Kiyoi and Dr. Yuichi Ishikawa from Nagoya University as co-authors, in addition to the original authors listed at the time of submission. Both Dr. Kiyoi and Dr. Ishikawa played pivotal roles in the analysis of cells from AML patients, significantly enhancing the quality of our research.
>
> We kindly request your concurrence with the inclusion of these esteemed colleagues as co-authors. Your approval would be greatly appreciated.If you concur with my suggestion, kindly respond with a straightforward statement, such as "I approve the change of the author list."
>
> Thank you sincerely for your cooperation.
>
> Warm regards,
>
> Atsushi Hirao
>
> --
> ----------------------
> Atsushi Hirao, M.D. Ph.D.

Professor
Division of Molecular Genetics
Cancer Research Institute
WPI Nano Life Science Institute(WPI-Nano LSI)
Kanazawa University

Kakuma-machi, Kanazawa, Ishikawa, Japan 920-1192
TEL +81-76-264-6755
FAX+81-76-234-4508
e-mail ahirao@staff.kanazawa-u.ac.jp

Subject: Re: Change of author list
From: jing y w <jingyw15@gmail.com>
Date: 2023/09/19 8:03
To: Atsushi Hirao <ahirao@staff.kanazawa-u.ac.jp>

Dear Dr. Hirao,

I approve the change of the author list.
Best,

Yongwei Jing

Atsushi Hirao <ahirao@staff.kanazawa-u.ac.jp>于2023年9月18日 周一16:10写道：

> Dear Co-Authors,
>
> I would like to extend our heartfelt gratitude for your invaluable contributions to the preparation of our paper titled, “Targeting cis-regulatory elements of FOXO family is a novel therapeutic strategy for induction of leukemia cell differentiation” for publication in Cell Death & Disease. In the process of revising the manuscript, we have included Dr. Hitoshi Kiyoi and Dr. Yuichi Ishikawa from Nagoya University as co-authors, in addition to the original authors listed at the time of submission. Both Dr. Kiyoi and Dr. Ishikawa played pivotal roles in the analysis of cells from AML patients, significantly enhancing the quality of our research.
>
> We kindly request your concurrence with the inclusion of these esteemed colleagues as co-authors. Your approval would be greatly appreciated.If you concur with my suggestion, kindly respond with a straightforward statement, such as "I approve the change of the author list."
>
> Thank you sincerely for your cooperation.
>
> Warm regards,
>
> Atsushi Hirao
>
> --
> -----------------------
> Atsushi Hirao, M.D. Ph.D.
>
> Professor
> Division of Molecular Genetics
> Cancer Research Institute
> WPI Nano Life Science Institute(WPI-Nano LSI)

Kanazawa University

Kakuma-machi, Kanazawa, Ishikawa, Japan 920-1192
TEL +81-76-264-6755
FAX+81-76-234-4508
e-mail ahirao@staff.kanazawa-u.ac.jp

Subject: Re: Change of author list
From: Atsuko Kasahara <akasahara@staff.kanazawa-u.ac.jp>
Date: 2023/09/19 10:10
To: Atsushi Hirao <ahirao@staff.kanazawa-u.ac.jp>

Dear Dr. Hirao,

I approve the change of the author list.
Best,
Atsuko Kasahara

> On Sep 18, 2023, at 16:09, Atsushi Hirao <ahirao@staff.kanazawa-u.ac.jp> wrote:
>
> Dear Co-Authors,
>
> I would like to extend our heartfelt gratitude for your invaluable contributions to the preparation of our paper titled, "Targeting cis-regulatory elements of FOXO family is a novel therapeutic strategy for induction of leukemia cell differentiation" for publication in Cell Death & Disease. In the process of revising the manuscript, we have included Dr. Hitoshi Kiyoi and Dr. Yuichi Ishikawa from Nagoya University as co-authors, in addition to the original authors listed at the time of submission. Both Dr. Kiyoi and Dr. Ishikawa played pivotal roles in the analysis of cells from AML patients, significantly enhancing the quality of our research.
>
> We kindly request your concurrence with the inclusion of these esteemed colleagues as co-authors. Your approval would be greatly appreciated.If you concur with my suggestion, kindly respond with a straightforward statement, such as "I approve the change of the author list."
>
> Thank you sincerely for your cooperation.
>
> Warm regards,
>
> Atsushi Hirao
>
> --
> -----------------------
> Atsushi Hirao, M.D. Ph.D.
>
> Professor
> Division of Molecular Genetics
> Cancer Research Institute
> WPI Nano Life Science Institute(WPI-Nano LSI)
> Kanazawa University
>
> Kakuma-machi, Kanazawa, Ishikawa, Japan 920-1192
> TEL +81-76-264-6755

> FAX+81-76-234-4508
> e-mail ahirao@staff.kanazawa-u.ac.jp

Atsuko KASAHARA, PhD,
Institute for Frontier Science Initiative,
Cancer Research Institute, Kanazawa University, Japan
+81762646795

Subject: Re: Change of author list
From: Hitoshi Kiyoi <kiyoi@med.nagoya-u.ac.jp>
Date: 2023/09/20 14:10
To: Atsushi Hirao <ahirao@staff.kanazawa-u.ac.jp>
CC: Hitoshi Kiyoi <kiyoi@med.nagoya-u.ac.jp>

Dear Dr. Hirao,

I approve the change of the author list.
Best,

Hitoshi Kiyoi

Hitoshi Kiyoi, MD, PhD.
Professor
Department of Hematology and Oncology,
Nagoya University Graduate School of Medicine.
65 Tsurumai-cho, Showa-ku,
Nagoya 466-8550, JAPAN.
Phone: +81-52-744-2136
FAX: +81-52-744-2157
E-mail: kiyoi@med.nagoya-u.ac.jp

> 2023/09/20 14:07、Atsushi Hirao <ahirao@staff.kanazawa-u.ac.jp>のメール:
>
> Dear Dear Dr. Kiyoi,
>
> I would like to extend our heartfelt gratitude for your invaluable contributions to the preparation of our paper titled, “Targeting cis-regulatory elements of FOXO family is a novel therapeutic strategy for induction of leukemia cell differentiation” for publication in Cell Death & Disease. In the process of revising the manuscript, we have included you and Dr. Yuichi Ishikawa from Nagoya University as co-authors, in addition to the original authors listed at the time of submission. Both you and Dr. Ishikawa played pivotal roles in the analysis of cells from AML patients, significantly enhancing the quality of our research.
>
> We kindly request your concurrence with the inclusion of these esteemed colleagues as co-authors. Your approval would be greatly appreciated.If you concur with my suggestion, kindly respond with a straightforward statement, such as "I approve the change of the author list."
>
> Thank you sincerely for your cooperation.
>
> Warm regards,
>
> Atsushi Hirao
>
> --

> -----------------------
> Atsushi Hirao, M.D. Ph.D.
>
> Professor
> Division of Molecular Genetics
> Cancer Research Institute
> WPI Nano Life Science Institute(WPI-Nano LSI)
> Kanazawa University
>
> Kakuma-machi, Kanazawa, Ishikawa, Japan 920-1192
> TEL +81-76-264-6755
> FAX+81-76-234-4508
> e-mail ahirao@staff.kanazawa-u.ac.jp

Subject: Re: Change of author list
From: 小林 昌彦 <mkoba@staff.kanazawa-u.ac.jp>
Date: 2023/09/19 14:06
To: Atsushi Hirao <ahirao@staff.kanazawa-u.ac.jp>

Dear Dr. Hirao,

I approve the change of the author list.
Best,

Masahiko Kobayashi

> 2023/09/18 16:09、Atsushi Hirao <ahirao@staff.kanazawa-u.ac.jp>のメール:
>
> Dear Co-Authors,
>
> I would like to extend our heartfelt gratitude for your invaluable contributions to the preparation of our paper titled, “Targeting cis-regulatory elements of FOXO family is a novel therapeutic strategy for induction of leukemia cell differentiation” for publication in Cell Death & Disease. In the process of revising the manuscript, we have included Dr. Hitoshi Kiyoi and Dr. Yuichi Ishikawa from Nagoya University as co-authors, in addition to the original authors listed at the time of submission. Both Dr. Kiyoi and Dr. Ishikawa played pivotal roles in the analysis of cells from AML patients, significantly enhancing the quality of our research.
>
> We kindly request your concurrence with the inclusion of these esteemed colleagues as co-authors. Your approval would be greatly appreciated.If you concur with my suggestion, kindly respond with a straightforward statement, such as "I approve the change of the author list."
>
> Thank you sincerely for your cooperation.
>
> Warm regards,
>
> Atsushi Hirao
>
> --
> -----------------------

> Atsushi Hirao, M.D. Ph.D.
>
> Professor
> Division of Molecular Genetics
> Cancer Research Institute
> WPI Nano Life Science Institute(WPI-Nano LSI)
> Kanazawa University
>
> Kakuma-machi, Kanazawa, Ishikawa, Japan 920-1192
> TEL +81-76-264-6755
> FAX+81-76-234-4508
> e-mail ahirao@staff.kanazawa-u.ac.jp

Subject: Re: Change of author list
From: kentakurayoshi@staff.kanazawa-u.ac.jp
Date: 2023/09/19 9:51
To: Atsushi Hirao <ahirao@staff.kanazawa-u.ac.jp>

Dear Dr. Hirao,

I approve the change of the author list.
Best,

Kenta Kurayoshi

2023-09-18 16:09 に Atsushi Hirao さんは書きました:

> Dear Co-Authors,
>
> I would like to extend our heartfelt gratitude for your invaluable contributions to the preparation of our paper titled, “Targeting cis-regulatory elements of FOXO family is a novel therapeutic strategy for induction of leukemia cell differentiation” for publication in Cell Death & Disease. In the process of revising the manuscript, we have included Dr. Hitoshi Kiyoi and Dr. Yuichi Ishikawa from Nagoya University as co-authors, in addition to the original authors listed at the time of submission. Both Dr. Kiyoi and Dr. Ishikawa played pivotal roles in the analysis of cells from AML patients, significantly enhancing the quality of our research.
>
> We kindly request your concurrence with the inclusion of these esteemed colleagues as co-authors. Your approval would be greatly appreciated.If you concur with my suggestion, kindly respond with a straightforward statement, such as "I approve the change of the author list."
>
> Thank you sincerely for your cooperation.
>
> Warm regards,
>
> Atsushi Hirao
>
> --
> -----------------------

> Atsushi Hirao, M.D. Ph.D.
>
> Professor
> Division of Molecular Genetics
> Cancer Research Institute
> WPI Nano Life Science Institute(WPI-Nano LSI)
> Kanazawa University
>
> Kakuma-machi, Kanazawa, Ishikawa, Japan 920-1192
> TEL +81-76-264-6755
> FAX+81-76-234-4508
> e-mail ahirao@staff.kanazawa-u.ac.jp

Subject: Re: Change of author list
From: Nagase Hiroki <nagasehiroki@hotmail.com>
Date: 2023/09/18 22:16
To: Atsushi Hirao <ahirao@staff.kanazawa-u.ac.jp>

Professor Hirao,

I approve the change of the author list.

Hiroki Nagase

差出人: Atsushi Hirao <ahirao@staff.kanazawa-u.ac.jp>
送信日時: 2023年9月18日 16:09
件名: Change of author list

Dear Co-Authors,

I would like to extend our heartfelt gratitude for your invaluable contributions to the preparation of our paper titled, "Targeting cis-regulatory elements of FOXO family is a novel therapeutic strategy for induction of leukemia cell differentiation" for publication in Cell Death & Disease. In the process of revising the manuscript, we have included Dr. Hitoshi Kiyoi and Dr. Yuichi Ishikawa from Nagoya University as co-authors, in addition to the original authors listed at the time of submission. Both Dr. Kiyoi and Dr. Ishikawa played pivotal roles in the analysis of cells from AML patients, significantly enhancing the quality of our research.

We kindly request your concurrence with the inclusion of these esteemed colleagues as co-authors. Your approval would be greatly appreciated.If you concur with my suggestion, kindly respond with a straightforward statement, such as "I approve the change of the author list."

Thank you sincerely for your cooperation.

Warm regards,

Atsushi Hirao

--

Atsushi Hirao, M.D. Ph.D.

Professor
Division of Molecular Genetics
Cancer Research Institute
WPI Nano Life Science Institute(WPI-Nano LSI)
Kanazawa University

Kakuma-machi, Kanazawa, Ishikawa, Japan 920-1192

TEL +81-76-264-6755
FAX+81-76-234-4508
e-mail ahirao@staff.kanazawa-u.ac.jp

Subject: Re: [EXTERNAL] Change of author list
From: "Nishida,Yuki" <YNishida@mdanderson.org>
Date: 2023/09/18 23:21
To: Atsushi Hirao <ahirao@staff.kanazawa-u.ac.jp>

Dear Dr. Hirao,

I approve the change of the author list.

Best,
Yuki

From: Atsushi Hirao <ahirao@staff.kanazawa-u.ac.jp>
Date: Monday, September 18, 2023 at 2:10 AM
To:
Subject: [EXTERNAL] Change of author list

THIS EMAIL IS A PHISHING RISK
Do you trust the sender?
The email address is: prvs=16282a44fc=ahirao@staff.kanazawa-u.ac.jp
While this email has passed our filters, we need you to review with caution before taking any action.
If the email looks at all suspicious, click the Report a Phish button.

Dear Co-Authors,

I would like to extend our heartfelt gratitude for your invaluable contributions to the preparation of our paper titled, "Targeting cis-regulatory elements of FOXO family is a novel therapeutic strategy for induction of leukemia cell differentiation" for publication in Cell Death & Disease. In the process of revising the manuscript, we have included Dr. Hitoshi Kiyoi and Dr. Yuichi Ishikawa from Nagoya University as co-authors, in addition to the original authors listed at the time of submission. Both Dr. Kiyoi and Dr. Ishikawa played pivotal roles in the analysis of cells from AML patients, significantly enhancing the quality of our research.

We kindly request your concurrence with the inclusion of these esteemed colleagues as co-authors. Your approval would be greatly appreciated.If you concur with my suggestion, kindly respond with a straightforward statement, such as "I approve the change of the author list."

Thank you sincerely for your cooperation.

Warm regards,

Atsushi Hirao

--

Atsushi Hirao, M.D. Ph.D.

Professor
Division of Molecular Genetics
Cancer Research Institute
WPI Nano Life Science Institute(WPI-Nano LSI)
Kanazawa University

Kakuma-machi, Kanazawa, Ishikawa, Japan 920-1192
TEL +81-76-264-6755
FAX+81-76-234-4508
e-mail ahirao@staff.kanazawa-u.ac.jp

The information contained in this e-mail message may be privileged, confidential, and/or protected from disclosure. This e-mail message may contain protected health information (PHI); dissemination of PHI should comply with applicable federal and state laws. If you are not the intended recipient, or an authorized representative of the intended recipient, any further review, disclosure, use, dissemination, distribution, or copying of this message or any attachment (or the information contained therein) is strictly prohibited. If you think that you have received this e-mail message in error, please notify the sender by return e-mail and delete all references to it and its contents from your systems.

Subject: RE: Change of author list
From: Kumiko Ohta <kumiohta@med.u-ryukyu.ac.jp>
Date: 2023/09/19 9:37
To: "'Atsushi Hirao'" <ahirao@staff.kanazawa-u.ac.jp>

Dear Dr. Hirao,

I approve the change of the author list.
Best,

Kumiko Ohta

-----Original Message-----
From: Atsushi Hirao <ahirao@staff.kanazawa-u.ac.jp>
Sent: Monday, September 18, 2023 4:10 PM
To: undisclosed-recipients:
Subject: Change of author list

Dear Co-Authors,

I would like to extend our heartfelt gratitude for your invaluable contributions to the preparation of our paper titled, "Targeting cis-regulatory elements of FOXO family is a novel therapeutic strategy for induction of leukemia cell differentiation" for publication in Cell Death & Disease. In the process of revising the manuscript, we have included Dr. Hitoshi Kiyoi and Dr. Yuichi Ishikawa from Nagoya University as co-authors, in addition to the original authors listed at the time of submission. Both Dr. Kiyoi and Dr. Ishikawa played pivotal roles in the analysis of cells from AML patients, significantly enhancing the quality of our research.

We kindly request your concurrence with the inclusion of these esteemed colleagues as co-authors. Your approval would be greatly appreciated.If you concur with my suggestion, kindly respond with a straightforward statement, such as "I approve the change of the author list."

Thank you sincerely for your cooperation.

Warm regards,

Atsushi Hirao

--

Atsushi Hirao, M.D. Ph.D.

Professor
Division of Molecular Genetics
Cancer Research Institute
WPI Nano Life Science Institute(WPI-Nano LSI) Kanazawa University

Kakuma-machi, Kanazawa, Ishikawa, Japan 920-1192 TEL +81-76-264-6755
FAX+81-76-234-4508
e-mail ahirao@staff.kanazawa-u.ac.jp

Subject: Re: Fwd: Change of author list
From: Mahmoud Shoulkamy <mahmoud@staff.kanazawa-u.ac.jp>
Date: 2023/09/20 14:48
To: Atsushi Hirao <ahirao@staff.kanazawa-u.ac.jp>

Dear Professor Hirao
Thank you so much for the email.
I approve the change of the author list.
I appreciate your kindness and nice cooperation.
Best regards
Mahmoud

On 20/09/2023 2:09 pm, Atsushi Hirao wrote:

> Dear Dr. Shoulkamy,
>
> I would like to extend our heartfelt gratitude for your invaluable contributions to the preparation of our paper titled, “Targeting cis-regulatory elements of FOXO family is a novel therapeutic strategy for induction of leukemia cell differentiation” for publication in Cell Death & Disease. In the process of revising the manuscript, we have included Dr. Hitoshi Kiyoi and Dr. Yuichi Ishikawa from Nagoya University as co-authors, in addition to the original authors listed at the time of submission. Both Dr. Kiyoi and Dr. Ishikawa played pivotal roles in the analysis of cells from AML patients, significantly enhancing the quality of our research.
>
> We kindly request your concurrence with the inclusion of these esteemed colleagues as co-authors. Your approval would be greatly appreciated.If you concur with my suggestion, kindly respond with a straightforward statement, such as "I approve the change of the author list."
>
> Thank you sincerely for your cooperation.
>
> Warm regards,
>
> Atsushi Hirao

Subject: Re: Change of author list
From: 杉山弘 <sugiyama.hiroshi.3s@kyoto-u.ac.jp>
Date: 2023/09/19 8:04
To: Atsushi Hirao <ahirao@staff.kanazawa-u.ac.jp>

Dear Dr. Hirao,

Thank you for your message.
I approve the change of the author list.

With best regards,

Hiroshi
--
Hiroshi Sugiyama,
Specially Appointed Professor,
http://kuchem.kyoto-u.ac.jp/chembio/publications.html
Institute for Integrated Cell-Material Sciences (WPI-iCeMS),
Kyoto University

2023年9月18日(月) 16:10 Atsushi Hirao <ahirao@staff.kanazawa-u.ac.jp>:

> Dear Co-Authors,
>
> I would like to extend our heartfelt gratitude for your invaluable contributions to the preparation of our paper titled, "Targeting cis-regulatory elements of FOXO family is a novel therapeutic strategy for induction of leukemia cell differentiation" for publication in Cell Death & Disease. In the process of revising the manuscript, we have included Dr. Hitoshi Kiyoi and Dr. Yuichi Ishikawa from Nagoya University as co-authors, in addition to the original authors listed at the time of submission. Both Dr. Kiyoi and Dr. Ishikawa played pivotal roles in the analysis of cells from AML patients, significantly enhancing the quality of our research.
>
> We kindly request your concurrence with the inclusion of these esteemed colleagues as co-authors. Your approval would be greatly appreciated.If you concur with my suggestion, kindly respond with a straightforward statement, such as "I approve the change of the author list."
>
> Thank you sincerely for your cooperation.
>
> Warm regards,
>
> Atsushi Hirao

--

Atsushi Hirao, M.D. Ph.D.

Professor
Division of Molecular Genetics
Cancer Research Institute
WPI Nano Life Science Institute(WPI-Nano LSI)
Kanazawa University

Kakuma-machi, Kanazawa, Ishikawa, Japan 920-1192
TEL +81-76-264-6755
FAX+81-76-234-4508
e-mail ahirao@staff.kanazawa-u.ac.jp

Subject: Re: Change of author list
From: 田嶋敦 <atajima@med.kanazawa-u.ac.jp>
Date: 2023/09/19 3:09
To: Atsushi Hirao <ahirao@staff.kanazawa-u.ac.jp>

Dear Prof. Hirao,

I approve the change of the author list.

Best regards,,

Atsushi Tajima

> 2023/09/18 9:09、Atsushi Hirao <ahirao@staff.kanazawa-u.ac.jp>のメール：
>
> Dear Co-Authors,
>
> I would like to extend our heartfelt gratitude for your invaluable contributions to the preparation of our paper titled, “Targeting cis-regulatory elements of FOXO family is a novel therapeutic strategy for induction of leukemia cell differentiation” for publication in Cell Death & Disease. In the process of revising the manuscript, we have included Dr. Hitoshi Kiyoi and Dr. Yuichi Ishikawa from Nagoya University as co-authors, in addition to the original authors listed at the time of submission. Both Dr. Kiyoi and Dr. Ishikawa played pivotal roles in the analysis of cells from AML patients, significantly enhancing the quality of our research.
>
> We kindly request your concurrence with the inclusion of these esteemed colleagues as co-authors. Your approval would be greatly appreciated.If you concur with my suggestion, kindly respond with a straightforward statement, such as "I approve the change of the author list."
>
> Thank you sincerely for your cooperation.
>
> Warm regards,
>
> Atsushi Hirao
>
> --
> -----------------------
> Atsushi Hirao, M.D. Ph.D.
>
> Professor
> Division of Molecular Genetics
> Cancer Research Institute
> WPI Nano Life Science Institute(WPI-Nano LSI)
> Kanazawa University
>
> Kakuma-machi, Kanazawa, Ishikawa, Japan 920-1192
> TEL +81-76-264-6755
> FAX+81-76-234-4508
> e-mail ahirao@staff.kanazawa-u.ac.jp

Atsushi Tajima, Ph.D.

Dean, Graduate School of Advanced Preventive Medical Sciences
Professor, Department of Bioinformatics and Genomics
Kanazawa University

13-1 Takara-machi, Kanazawa
Ishikawa 920-8640, JAPAN
Phone: +81-76-265-2715
Fax: +81-76-234-4249
E-mail: atajima@med.kanazawa-u.ac.jp
Lab-HP: https://big.w3.kanazawa-u.ac.jp/

Subject: Re: Change of author list
From: Yusuke Takase <takaseyusuke@gmail.com>
Date: 2023/09/19 8:24
To: Atsushi Hirao <ahirao@staff.kanazawa-u.ac.jp>

Dear Dr. Hirao,

I approve the change of the author list.
Best,

Yusuke Takase

2023年9月18日(月) 16:10 Atsushi Hirao <ahirao@staff.kanazawa-u.ac.jp>:

> Dear Co-Authors,
>
> I would like to extend our heartfelt gratitude for your invaluable contributions to the preparation of our paper titled, "Targeting cis-regulatory elements of FOXO family is a novel therapeutic strategy for induction of leukemia cell differentiation" for publication in Cell Death & Disease. In the process of revising the manuscript, we have included Dr. Hitoshi Kiyoi and Dr. Yuichi Ishikawa from Nagoya University as co-authors, in addition to the original authors listed at the time of submission. Both Dr. Kiyoi and Dr. Ishikawa played pivotal roles in the analysis of cells from AML patients, significantly enhancing the quality of our research.
>
> We kindly request your concurrence with the inclusion of these esteemed colleagues as co-authors. Your approval would be greatly appreciated.If you concur with my suggestion, kindly respond with a straightforward statement, such as "I approve the change of the author list."
>
> Thank you sincerely for your cooperation.
>
> Warm regards,
>
> Atsushi Hirao
>
> --
> -----------------------
> Atsushi Hirao, M.D. Ph.D.
>
> Professor
> Division of Molecular Genetics
> Cancer Research Institute
> WPI Nano Life Science Institute(WPI-Nano LSI)

Kanazawa University

Kakuma-machi, Kanazawa, Ishikawa, Japan 920-1192
TEL +81-76-264-6755
FAX+81-76-234-4508
e-mail ahirao@staff.kanazawa-u.ac.jp

Subject: Re: Change of author list
From: Masaya Ueno <masayaueno@staff.kanazawa-u.ac.jp>
Date: 2023/09/19 9:58
To: Atsushi Hirao <ahirao@staff.kanazawa-u.ac.jp>

Dear Dr. Hirao,

I approve the change of the author list.
Best,

Masaya Ueno

> 2023/09/18 16:09、Atsushi Hirao <ahirao@staff.kanazawa-u.ac.jp>のメール:
>
> Dear Co-Authors,
>
> I would like to extend our heartfelt gratitude for your invaluable contributions to the preparation of our paper titled, “Targeting cis-regulatory elements of FOXO family is a novel therapeutic strategy for induction of leukemia cell differentiation” for publication in Cell Death & Disease. In the process of revising the manuscript, we have included Dr. Hitoshi Kiyoi and Dr. Yuichi Ishikawa from Nagoya University as co-authors, in addition to the original authors listed at the time of submission. Both Dr. Kiyoi and Dr. Ishikawa played pivotal roles in the analysis of cells from AML patients, significantly enhancing the quality of our research.
>
> We kindly request your concurrence with the inclusion of these esteemed colleagues as co-authors. Your approval would be greatly appreciated.If you concur with my suggestion, kindly respond with a straightforward statement, such as "I approve the change of the author list."
>
> Thank you sincerely for your cooperation.
>
> Warm regards,
>
> Atsushi Hirao
>
> --
> -----------------------

> Atsushi Hirao, M.D. Ph.D.
>
> Professor
> Division of Molecular Genetics
> Cancer Research Institute
> WPI Nano Life Science Institute(WPI-Nano LSI)
> Kanazawa University
>
> Kakuma-machi, Kanazawa, Ishikawa, Japan 920-1192
> TEL +81-76-264-6755
> FAX+81-76-234-4508
> e-mail ahirao@staff.kanazawa-u.ac.jp

Subject: Re: Change of author list
From: khosomic 細道 一善 <khosomic@toyaku.ac.jp>
Date: 2023/09/18 16:21
To: Atsushi Hirao <ahirao@staff.kanazawa-u.ac.jp>

Dear Dr. Hirao,

I approve the change of the author list.
Best,

Kazuyoshi Hosomichi

===
Kazuyoshi Hosomichi, Ph.D.
Professor
Laboratory of Computational Genomics, School of Life Science
Tokyo University of Pharmacy and Life Sciences
1432-1 Horinouchi, Hachioji
Tokyo 192-0392
Japan
e-mail: khosomic@toyaku.ac.jp <mailto:khosomic@toyaku.ac.jp>
TEL: +81 42 676 7214

2023/09/18 16:10 に、"Atsushi Hirao" <ahirao@staff.kanazawa-u.ac.jp <mailto:ahirao@staff.kanazawa-u.ac.jp>> を書き込みました:

Dear Co-Authors,

I would like to extend our heartfelt gratitude for your invaluable contributions to the preparation of our paper titled, “Targeting cis-regulatory elements of FOXO family is a novel therapeutic strategy for induction of leukemia cell differentiation” for publication in Cell Death & Disease. In the process of revising the manuscript, we have included Dr. Hitoshi Kiyoi and Dr. Yuichi Ishikawa from Nagoya University as co-authors, in addition to the original authors listed at the time of submission. Both Dr. Kiyoi and Dr. Ishikawa played pivotal roles in the analysis of cells from AML patients, significantly enhancing the quality of our research.

We kindly request your concurrence with the inclusion of these esteemed colleagues as co-authors. Your approval would be greatly appreciated.If you concur with my suggestion, kindly respond with a straightforward statement, such as "I approve the change of the author list."

Thank you sincerely for your cooperation.

Warm regards,

Atsushi Hirao

--

Atsushi Hirao, M.D. Ph.D.

Professor
Division of Molecular Genetics
Cancer Research Institute
WPI Nano Life Science Institute(WPI-Nano LSI)
Kanazawa University

Kakuma-machi, Kanazawa, Ishikawa, Japan 920-1192
TEL +81-76-264-6755
FAX+81-76-234-4508
e-mail ahirao@staff.kanazawa-u.ac.jp <mailto:ahirao@staff.kanazawa-u.ac.jp>

Subject: Re: Change of author list
From: 小島 研介 <k-koji@kochi-u.ac.jp>
Date: 2023/09/18 16:12
To: 平尾 敦先生 金沢大学がん進展制御研究所： <ahirao@staff.kanazawa-u.ac.jp>

Dear Dr. Hirao,

I approve the change of the author list.

Sincerely,

Kensuke Kojima

> On Sep 18, 2023, at 16:09, Atsushi Hirao <ahirao@staff.kanazawa-u.ac.jp> wrote:
>
> Dear Co-Authors,
>
> I would like to extend our heartfelt gratitude for your invaluable contributions to the preparation of our paper titled, “Targeting cis-regulatory elements of FOXO family is a novel therapeutic strategy for induction of leukemia cell differentiation” for publication in Cell Death & Disease. In the process of revising the manuscript, we have included Dr. Hitoshi Kiyoi and Dr. Yuichi Ishikawa from Nagoya University as co-authors, in addition to the original authors listed at the time of submission. Both Dr. Kiyoi and Dr. Ishikawa played pivotal roles in the analysis of cells from AML patients, significantly enhancing the quality of our research.
>
> We kindly request your concurrence with the inclusion of these esteemed colleagues as co-authors. Your approval would be greatly appreciated.If you concur with my suggestion, kindly respond with a straightforward statement, such as "I approve the change of the author list."
>
> Thank you sincerely for your cooperation.
>
> Warm regards,
>
> Atsushi Hirao
>
> --
> ------------------------
> Atsushi Hirao, M.D. Ph.D.
>
> Professor
> Division of Molecular Genetics
> Cancer Research Institute
> WPI Nano Life Science Institute(WPI-Nano LSI)
> Kanazawa University
>
> Kakuma-machi, Kanazawa, Ishikawa, Japan 920-1192
> TEL +81-76-264-6755
> FAX+81-76-234-4508
> e-mail ahirao@staff.kanazawa-u.ac.jp

Subject: Re: Change of author list
From: Shuji Ikeda <s-ik@kc5.so-net.ne.jp>
Date: 2023/09/18 17:21
To: Atsushi Hirao <ahirao@staff.kanazawa-u.ac.jp>

Dear Dr. Hirao,

I approve the change of the author list.

Best,
Shuji Ikeda

On 2023/09/18 16:09, Atsushi Hirao wrote:

> Dear Co-Authors,
>
> I would like to extend our heartfelt gratitude for your invaluable contributions to the preparation of our paper titled, “Targeting cis-regulatory elements of FOXO family is a novel therapeutic strategy for induction of leukemia cell differentiation” for publication in Cell Death & Disease. In the process of revising the manuscript, we have included Dr. Hitoshi Kiyoi and Dr. Yuichi Ishikawa from Nagoya University as co-authors, in addition to the original authors listed at the time of submission. Both Dr. Kiyoi and Dr. Ishikawa played pivotal roles in the analysis of cells from AML patients, significantly enhancing the quality of our research.
>
> We kindly request your concurrence with the inclusion of these esteemed colleagues as co-authors. Your approval would be greatly appreciated.If you concur with my suggestion, kindly respond with a straightforward statement, such as "I approve the change of the author list."
>
> Thank you sincerely for your cooperation.
>
> Warm regards,
>
> Atsushi Hirao

Subject: Re: Change of author list
From: 田所優子 <tadokoro@staff.kanazawa-u.ac.jp>
Date: 2023/09/18 16:25
To: Atsushi Hirao <ahirao@staff.kanazawa-u.ac.jp>

Dear Dr. Hirao,

I approve the change of the author list.

Best,

Yuko Tadokoro

On 2023/09/18 16:09, Atsushi Hirao wrote:

> Dear Co-Authors,
>
> I would like to extend our heartfelt gratitude for your invaluable contributions to the preparation of our paper titled, “Targeting cis-regulatory elements of FOXO family is a novel therapeutic strategy for induction of leukemia cell differentiation” for publication in Cell Death & Disease. In the process of revising the manuscript, we have included Dr. Hitoshi Kiyoi and Dr. Yuichi Ishikawa from Nagoya University as co-authors, in addition to the original authors listed at the time of submission. Both Dr. Kiyoi and Dr. Ishikawa played pivotal roles in the analysis of cells from AML patients, significantly enhancing the quality of our research.
>
> We kindly request your concurrence with the inclusion of these esteemed colleagues as co-authors. Your approval would be greatly appreciated.If you concur with my suggestion, kindly respond with a straightforward statement, such as "I approve the change of the author list."
>
> Thank you sincerely for your cooperation.
>
> Warm regards,
>
> Atsushi Hirao

Subject: Re: Change of author list
From: "T.Watanabe" <twatanabe@chiba-cc.jp>
Date: 2023/09/20 14:35
To: Atsushi Hirao <ahirao@staff.kanazawa-u.ac.jp>

Dear Dr. Hirao,

I approve the change of the author list.
Best,

[Takayoshi Watanabe]
--
〒260-8717
千葉県千葉市中央区仁戸名町666-2
千葉県がんセンター研究所　発がん制御研究部
渡部　隆義
E-mail: twatanabe@chiba-cc.jp
Tel: 043-264-5431（内線8516）
